# Supplementary material for: Phylogenetic Aspects of Higher Plant Lipid Fatty Acid Profile
Source: Int J Mol Sci. 2025 Sep 26;26(19):9424. doi: 10.3390/ijms26199424 (PMC12524838; doi:10.3390/ijms26199424)
Supplement: Supplementary file 1 [file ijms-26-09424-s001.zip › Table_S1.pdf]

Table S1. Fatty acid profile of Embriophyta species (% of fatty acids methyl ester)

Species	Number	4:0	6:0	8:0	9:0	Unidentified shot chain	10:0	11:0	11:1(10)	12:00	12:1(7)	12:1(9)	13:0	13:1(8)	13:1(11)	14:0	14:1(7)	14:1(9)	14:2(6,9)	15:0	15:1(5)	15:1(6)	15:1(7)	15:1(8)	15:1(9)	15:1(10)	16:0	16:1(3)	16:1(6)
Carissa edulis [119]	1	-	-	-	-	-	-	-	-	-	-	-	-	-	-	0.1	-	-	-	-	-	-	-	-	-	-	21.0	-	-
Lomelosia cretica [112]	2	-	-	-	-	-	-	-	-	-	-	-	-	-	-	1.6	-	-	-	0.6	-	-	-	-	-	-	23.3	-	-
Aristolochia fontanesii [121]	3	-	-	-	-	-	2.2	-	-	3.9	-	-	-	-	-	2.5	-	-	-	-	-	-	-	-	-	-	15.6	-	-
Strelitzia reginae [122]	4	-	-	-	-	-	tr.	-	-	0.1	-	-	-	-	-	0.3	-	-	-	-	-	-	-	-	-	-	27.2	-	-
Cistus ladanifer [123]	5	-	-	-	-	-	-	-	-	0.2	-	-	-	-	-	2.1	-	-	-	-	-	-	-	-	-	-	13.4	-	-
Abelmoschus esculentus [119]	6	-	-	-	-	-	-	-	-	-	-	-	-	-	-	tr.	-	-	-	-	-	-	-	-	-	-	41.8	-	-
Malvella sherardiana [124]	7	-	-	-	-	-	-	-	-	0.9	-	-	-	-	-	2.9	-	-	-	-	-	-	-	-	-	-	25.6	-	-
Hibiscus cannabinus [125]	8	-	-	-	-	-	-	-	-	-	-	-	-	-	-	1.6	-	-	-	0.2	-	-	-	-	-	-	18.7	-	-
Hibiscus sabdariffa [124]	9	-	-	-	-	-	-	-	-	-	-	-	-	-	-	2.0	-	-	-	-	-	-	-	-	-	-	16.8	-	-
Malva neglecta [124]	10	-	-	-	-	-	-	-	1.6	-	-	-	-	-	-	7.8	-	-	-	-	-	-	-	-	-	-	36.8	-	2.3
Malva sylvestris [126]	11	-	tr.	tr.	-	-	tr.	-	-	0.1	-	-	-	-	-	0.5	-	0.2	-	0.1	-	-	-	-	-	-	9.8	-	-
Glyphaea brevis [127]	12	-	-	-	-	-	-	-	-	-	-	-	-	-	-	2.6	-	-	-	-	-	-	-	-	-	-	22.4	-	-
Corchorus olitorius [125]	13	-	-	-	-	-	-	-	-	-	-	-	-	-	-	2.1	-	0.2	-	-	-	-	-	-	-	-	20.2	-	-
Adansonia digitata [119]	14	-	-	-	-	-	-	-	-	-	-	-	-	-	-	0.2	-	-	-	-	-	-	-	-	-	-	45.5	-	-
Sterculia tragacantha [128]	15	-	-	-	-	-	-	-	-	0.6	-	-	0.2	-	-	1.1	-	1.1	-	0.2	-	-	-	-	-	1.5	33.4	-	-
Dioscorea communis [129]	16	-	-	0.1	-	-	tr.	-	-	0.1	-	-	tr.	-	-	0.3	-	tr.	-	0.2	-	-	-	-	-	-	14.9	-	-
Raphia sudanica [130]	17	-	-	-	-	-	7.4	-	-	tr.	-	-	-	-	-	4.5	-	0.6	-	tr.	-	-	-	-	-	-	18.6	-	-
Raphia regalis [130]	18	-	-	-	-	-	0.4	-	-	0.4	-	-	-	-	-	0.6	-	0.5	-	tr.	-	-	-	-	-	-	26.1	-	-
Raphia vinifera [130]	19	-	-	-	-	-	4.2	-	-	tr.	-	-	-	-	-	3.5	-	0.7	-	tr.	-	-	-	-	-	-	28.2	-	-
Raphia hookeri [130]	20	-	-	-	-	-	8.6	-	-	tr.	-	-	-	-	-	13.1	-	0.6	-	tr.	-	-	-	-	-	-	22.1	-	-
Elaeis guineensis [131]	21	-	-	-	-	-	-	-	-	0.2	-	-	-	-	-	0.5	-	-	-	-	-	-	-	-	-	-	21.7	-	-
Papaver rhoeas [120]	22	-	-	-	-	-	-	-	-	tr.	-	-	-	-	-	2.5	-	-	-	0.5	-	-	-	-	3.2	-	20.7	-	-
Vitis vinifera [132]	23	-	-	0.1	-	-	0.2	-	-	1.4	-	-	0.1	-	-	2.9	-	tr.	-	1.4	-	-	-	-	-	0.4	24.2	-	-
Arum elongatum [133]	24	-	0.1	0.1	-	-	0.1	-	-	0.3	-	-	-	-	-	0.4	-	-	-	-	-	-	-	-	-	-	15.8	-	-
Xanthosoma sagittifolium [125]	25	-	-	-	-	-	-	-	-	-	-	-	-	-	-	1.7	-	-	-	-	-	-	-	-	-	-	24.1	-	-
Pleroma granulosum [134]	26	-	-	-	-	-	-	-	-	-	-	-	-	-	-	-	-	-	-	-	-	-	-	-	-	-	22.6	-	-
Lumnitzera racemosa [135]	27	-	-	-	-	-	-	-	-	9.0	-	-	0.7	-	-	7.9	-	-	-	1.5	-	-	-	-	-	-	61.5	-	-
Eucalyptus accedens [136]	28	-	-	-	-	-	-	-	-	-	-	-	-	-	-	0.8	-	-	-	-	-	-	-	-	-	-	22.7	-	-
Eucalyptus patens [136]	29	-	-	-	-	-	-	-	-	-	-	-	-	-	-	0.4	-	-	-	-	-	-	-	-	-	-	22.9	-	-
Eucalyptus wandoo [136]	30	-	-	-	-	-	-	-	-	1.1	-	-	-	-	-	0.9	-	-	-	-	-	-	-	-	-	-	24.5	-	-
Eucalyptus marginata [136]	31	-	-	-	-	-	-	-	-	-	-	-	-	-	-	0.6	-	-	-	-	-	-	-	-	-	-	29.6	-	-
Eucalyptus diversicolor [136]	32	-	-	-	-	-	-	-	-	-	-	-	-	-	-	0.4	-	-	-	-	-	-	-	-	-	-	19.1	-	-
Myrtus communis [137]	33	-	-	-	-	-	-	-	-	4.3	-	-	-	-	-	3.0	-	-	-	0.5	-	-	-	-	-	-	15.7	-	-
Corymbia calophylla [136]	34	-	-	-	-	-	-	-	-	1.5	-	-	-	-	-	2.9	-	-	-	-	-	-	-	-	-	-	17.4	-	-
Punica granatum [132]	35	-	-	0.4	-	-	0.8	0.9	-	0.8	-	-	0.3	-	-	2.0	-	0.5	-	0.2	-	-	-	-	-	1.7	19.9	-	-
Juglans regia [132]	36	-	-	0.1	-	-	0.1	0.1	-	1.7	-	-	tr.	-	-	2.0	-	0.1	-	0.4	-	-	-	-	-	1.5	16.9	-	-
Quercus ilex [138]	37	-	-	-	-	-	-	-	-	-	-	-	-	-	-	0.4	-	-	-	-	-	-	-	-	-	-	9.2	-	-
Castanea sativa [139]	38	-	0.2	0.5	-	-	0.3	-	-	0.3	-	-	tr.	-	-	1.1	-	0.2	-	0.3	-	-	-	-	-	-	14.4	-	-
Cynoglossum creticum [120]	39	-	-	-	-	-	-	-	-	-	-	-	-	-	-	1.2	-	-	-	-	-	-	-	-	1.0	-	16.1	-	-
Anchusa azurea [129]	40	-	-	-	-	-	0.1	tr.	-	0.1	-	-	tr.	-	-	0.4	-	0.2	-	0.1	-	-	-	-	-	-	10.5	-	-
Anchusa officinalis [133]	41	-	0.1	0.1	-	-	0.4	-	-	0.3	-	-	-	-	-	1.2	-	-	-	-	-	-	-	-	-	-	36.1	-	-
Borago officinalis [140]	42	-	-	-	-	-	-	-	-	-	-	-	-	-	-	-	-	-	-	-	-	-	-	-	-	-	16.4	-	-
Lilium longiflorum *	43	-	-	-	-	-	-	-	-	0.2	-	-	-	-	-	0.6	-	-	-	0.2	-	-	-	-	-	-	19.6	-	-
Asparagus acutifolius [129]	44	-	-	0.2	-	-	0.4	-	-	0.5	-	-	-	-	-	0.7	-	-	-	0.6	-	-	-	-	-	-	28.7	-	-
Xanthorrhoea preissii [122]	45	-	-	-	-	-	-	-	-	0.6	-	-	-	-	-	tr.	-	-	-	-	-	-	-	-	-	-	6.4	-	-
Eremurus spectabilis [133]	46	-	-	0.3	-	-	0.1	-	-	0.9	-	-	-	-	-	1.3	-	-	-	-	-	-	-	-	-	-	37.8	-	-
Muscari comosum [120]	47	-	-	-	-	-	-	-	-	-	-	-	-	-	-	-	-	-	-	-	-	-	-	-	-	-	18.2	-	-
Allium orientale [133]	48	-	0.2	0.3	-	-	0.2	-	-	0.6	-	-	-	-	-	1.0	-	-	-	-	-	-	-	-	-	-	33.7	-	-
Allium schoenoprasum [120]	49	-	-	-	-	-	-	-	-	-	-	-	-	-	-	0.3	-	-	-	-	-	-	-	-	-	-	14.1	-	-
Allium sativum [133]	50	-	-	-	-	-	-	-	-	-	-	-	-	-	-	-	-	0.3	-	-	-	-	-	-	-	-	20.0	-	-
Allium ampeloprasum [141]	51	-	-	-	-	-	0.2	-	-	-	-	-	-	-	-	-	-	0.2	-	0.2	-	-	-	-	-	-	0.2	-	-
Allium cepa [141]	52	-	-	-	-	-	0.6	-	-	-	-	-	-	-	-	-	-	-	-	0.8	-	-	-	-	-	-	20.4	-	-
Carex depressa [142]	53	-	-	-	-	-	-	-	-	-	-	-	-	-	-	2.4	-	-	-	-	-	-	-	-	-	-	59.8	-	-
Carex cilicica [142]	54	-	-	-	-	-	-	-	-	-	-	-	-	-	-	2.9	-	-	-	-	-	-	-	-	-	-	54.6	-	-
Carex halleriana [142]	55	-	-	-	-	-	-	-	-	-	-	-	-	-	-	2.5	-	-	-	-	-	-	-	-	-	-	53.0	-	-
Carex hartmanii [142]	56	-	-	-	-	-	-	-	-	-	-	-	-	-	-	3.5	-	-	-	-	-	-	-	-	-	-	78.4	-	-
Carex orbicularis [142]	57	-	-	-	-	-	-	-	-	-	-	-	-	-	-	2.2	-	-	-	-	-	-	-	-	-	-	70.0	-	-
Carex punctata [142]	58	-	-	-	-	-	-	-	-	-	-	-	-	-	-	3.9	-	-	-	-	-	-	-	-	-	-	70.5	-	-
Carex pendula [142]	59	-	-	-	-	-	-	-	-	-	-	-	-	-	-	3.3	-	-	-	-	-	-	-	-	-	-	59.1	-	-
Carex sylvatica [142]	60	-	-	-	-	-	-	-	-	-	-	-	-	-	-	2.7	-	-	-	-	-	-	-	-	-	-	72.4	-	-
Carex rostrata [142]	61	-	-	-	-	-	-	-	-	-	-	-	-	-	-	3.2	-	-	-	-	-	-	-	-	-	-	77.6	-	-
Carex liparocarpus [142]	62	-	-	-	-	-	-	-	-	-	-	-	-	-	-	2.0	-	-	-	-	-	-	-	-	-	-	51.9	-	-
Carex paniculata [142]	63	-	-	-	-	-	-	-	-	-	-	-	-	-	-	4.9	-	-	-	-	-	-	-	-	-	-	65.8	-	-
Carex pallescens [142]	64	-	-	-	-	-	-	-	-	-	-	-	-	-	-	2.1	-	-	-	-	-	-	-	-	-	-	63.8	-	-
Carex flacca [142]	65	-	-	-	-	-	-	-	-	-	-	-	-	-	-	3.4	-	-	-	-	-	-	-	-	-	-	62.7	-	-
Carex digitata [142]	66	-	-	-	-	-	-	-	-	-	-	-	-	-	-	2.3	-	-	-	-	-	-	-	-	-	-	64.0	-	-
Carex brevicollis [142]	67	-	-	-	-	-	-	-	-	-	-	-	-	-	-	2.0	-	-	-	-	-	-	-	-	-	-	56.2	-	-
Zea mays [143]	68	-	-	-	-	-	-	-	-	5.7	-	-	-	-	-	39.9	-	-	-	-	-	-	-	-	-	-	-	-	-
Leucopogon conostephioides [122]	69	-	-	-	-	-	-	-	-	-	-	-	-	-	-	0.3	-	-	-	-	-	-	-	-	-	-	23.3	-	-
Vaccinium myrtillus [144]	70	-	-	-	-	-	-	-	-	-	-	-	-	-	-	-	-	-	-	1.1	-	-	-	-	-	-	21.1	-	-
Arbutus unedo [145]	71	-	-	-	-	-	-	-	-	-	-	-	-	-	-	5.3	-	-	-	-	-	-	-	-	-	-	25.5	-	-
Arbutus andrachne [145]	72	-	-	-	-	-	-	-	-	-	-	-	-	-	-	3.0	-	-	-	-	-	-	-	-	-	-	22.6	-	-
Labisia pumila var. pumila [146]	73	-	-	-	-	-	-	-	-	-	-	-	-	-	-	-	-	-	-	-	-	-	-	-	-	-	27.1	-	-
Labisia pumila var. alata [146]	74	-	-	-	-	-	-	-	-	-	-	-	-	-	-	-	-	-	-	-	-	-	-	-	-	-	28.2	-	-
Labisia pumila var. lanceolata [146]	75	-	-	-	-	-	-	-	-	-	-	-	-	-	-	-	-	-	-	-	-	-	-	-	-	-	24.6	-	-
Aegiceras corniculatum [135]	76	-	-	-	-	-	-	-	-	37.8	-	-	-	-	-	2.2	-	-	-	0.5	-	-	-	-	-	-	34.1	-	-
Camellia Sinensis [147]	77	-	-	0.5	-	-	0.6	-	-	4.5	-	-	-	-	-	3.2	-	-	-	-	-	-	-	-	-	-	29.8	-	-
Eucommia ulmoides [148]	78	-	-	tr.	-	-	tr.	tr.	-	tr.	-	-	tr.	-	-	0.5	-	-	-	tr.	-	-	-	-	-	tr.	29.8	-	-
Artemisia lercheana [16]	79	-	-	-	-	-	-	-	-	-	-	-	-	-	-	1.5	-	-	-	-	-	-	-	-	-	-	14.5	-	-
Tragopogon sinuatus [120]	80	-	-	-	-	-	-	-	-	-	-	-	-	-	-	2.1	-	0.1	-	0.2	-	-	-	-	2.7	-	18.0	-	-
Crepis vesicaria [120]	81	-	-	-	-	-	-	-	-	-	-	-	-	-	-	1.8	-	-	-	0.3	-	-	-	-	2.2	-	20.8	-	-
Helichrysum stoechas [139]	82	-	0.2	2.4	-	-	1.7	0.5	-	7.6	-	-	0.2	-	-	7.9	-	0.2	-	0.6	-	-	-	-	-	-	13.2	-	-
Arctotheca calendula [122]	83	-	-	-	-	-	-	-	-	3.1	-	-	-	-	-	tr.	-	-	-	-	-	-	-	-	-	-	13.1	-	-
Symphyotrichum novi-belgii [122]	84	-	-	-	-	-	-	-	-	0.7	-	-	-	-	-	4.7	-	-	-	-	-	-	-	-	-	-	27.5	-	-
Helminthotheca echioides [122]	85	-	-	-	-	-	-	-	-	8.4	-	-	-	-	-	1.2	-	-	-	-	-	-	-	-	-	-	11.0	-	-
Gundelia tournefortii [149]	86	-	-	-	-	-	-	-	-	-	-	-	-	-	-	-	-	-	-	-	-	-	-	-	-	-	8.1	-	-
Stizolophus balsamita [150]	87	-	-	0.1	-	-	0.1	tr.	-	0.8	-	-	0.1	-	-	1.7	-	tr.	-	0.3	-	-	-	-	0.1	-	24.2	-	-
Matricaria chamomilla var. recutita [139]	88	-	0.2	0.2	-	-	0.2	0.1	-	0.2	-	-	tr.	-	-	1.3	-	0.2	-	0.3	-	-	-	-	-	-	13.6	-	-
Urospermum dalechampii [122]	89	-	-	-	-	-	-	-	-	6.4	-	-	-	-	-	3.5	-	-	-	-	-	-	-	-	-	-	20.8	-	-
Scolymus hispanicus [120]	90	-	-	-	-	-	-	-	-	-	-	-	-	-	-	1.0	-	-	-	0.8	-	-	-	-	-	-	25.2	-	-
Dahlia variabilis [120]	91	-	0.9	0.9	-	-	1.0	-	-	0.7	-	-	-	-	-	3.1	-	0.6	-	0.7	-	-	-	-	-	-	24.6	-	-
Silybum marianum [129]	92	-	-	-	-	-	0.2	-	-	0.5	-	-	-	-	-	1.0	-	-	-	1.1	-	-	-	-	-	-	28.7	-	-
Gymnanthemum amygdalinum [151]	93	-	-	-	-	-	-	-	-	-	-	0.9	-	-	-	1.2	-	-	-	-	-	-	-	-	-	-	22.2	-	-
Rhanterium adpressum [152]	94	-	-	-	-	-	-	-	-	1.9	-	-	-	-	-	4.0	-	-	-	0.7	-	-	-	-	-	-	47.4	-	-
Chondrilla juncea [129]	95	-	tr.	tr.	-	-	0.1	tr.	-	0.1	-	-	-	-	-	0.6	-	-	-	0.2	-	-	-	-	-	-	13.0	-	-
Hypochaeris cretensis [120]	96	-	-	-	-	-	-	-	-	-	-	-	-	-	-	2.4	-	-	-	0.4	-	-	-	-	2.8	-	20.6	-	-
Telekia speciosa [153]	97	-	-	-	-	-	-	-	-	-	-	-	-	-	-	-	-	-	-	-	-	-	-	-	-	-	20.3	-	-
Dittrichia viscosa [154]	98	-	-	-	-	-	-	-	-	-	-	-	-	-	-	1.4	-	-	-	-	-	-	-	-	-	-	19.0	-	-
Stevia rebaudiana [155]	99	-	-	-	-	-	-	-	-	-	-	-	-	-	-	-	-	-	-	-	-	-	-	-	-	-	27.5	-	-
Taraxacum obovatum [129]	100	-	-	0.1	-	-	0.1	tr.	-	0.4	-	-	-	-	-	0.8	-	0.3	-	0.3	-	-	-	-	-	-	11.8	-	-
Taraxacum officinale [144]	101	-	-	-	-	-	-	-	-	0.6	-	-	-	-	-	2.2	-	-	-	1.1	-	-	-	-	-	-	27.0	-	-
Sonchus oleraceus [129]	102	-	0.4	0.1	-	-	0.1	-	-	0.2	-	-	-	-	-	1.5	-	-	-	1.0	-	-	-	-	-	-	10.4	-	-
Reichardia picroides [120]	103	-	-	-	-	-	-	-	-	-	-	-	-	-	-	2.2	-	-	-	-	-	-	-	-	-	-	21.1	-	-
Centaurea pulcherrima [156]	104	-	-	tr.	-	-	0.8	0.1	-	1.1	-	-	tr.	-	-	0.9	-	tr.	-	0.5	-	-	-	-	-	1.2	23.4	-	-
Centaurea pseudoscabiosa [156]	105	-	-	tr.	-	-	0.1	0.1	-	1.7	-	-	0.2	-	-	0.9	-	tr.	-	0.2	-	-	-	-	-	0.7	25.8	-	-
Centaurea triumfettii [157]	106	-	-	tr.	-	-	0.1	tr.	-	0.3	-	-	tr.	-	-	2.0	-	0.1	-	0.2	-	-	-	-	0.1	-	18.3	-	-
Centaurea tchihatcheffii [158]	107	-	-	0.5	-	-	0.2	0.5	-	1.8	-	-	0.3	-	-	1.1	-	0.2	-	1.4	-	-	-	-	0.3	-	13.2	-	-
Centaurea saligna [159]	108	-	0.2	0.5	-	-	-	-	-	0.4	-	-	-	-	-	0.5	-	-	-	-	-	-	-	-	-	-	16.6	-	-
Centaurea iberica [159]	109	-	-	0.1	-	-	tr.	0.2	-	1.7	-	-	0.1	-	-	1.5	-	tr.	-	0.3	-	-	-	-	0.1	-	23.1	-	-
Centaurea calolepis [150]	110	-	-	0.1	-	-	0.1	tr.	-	1.1	-	-	0.1	-	-	2.5	-	tr.	-	0.4	-	-	-	-	0.1	-	20.2	-	-
Centaurea babylonica [156]	111	-	-	tr.	-	-	0.1	0.16	-	1.8	-	-	0.1	-	-	0.8	-	tr.	-	0.5	-	-	-	-	-	1.2	30.5	-	-
Centaurea solstitialis [157]	112	-	-	0.2	-	-	0.1	tr.	-	1.1	-	-	tr.	-	-	3.2	-	tr.	-	0.4	-	-	-	-	0.1	-	27.2	-	-
Centaurea carduiformis [150]	113	-	-	0.1	-	-	tr.	tr.	-	1.9	-	-	0.1	-	-	3.8	-	tr.	-	0.3	-	-	-	-	0.1	-	17.8	-	-
Centaurea virgata [157]	114	-	-	0.9	-	-	0.4	0.3	-	1.9	-	-	tr.	-	-	1.2	-	0.1	-	0.4	-	-	-	-	0.1	-	28.4	-	-
Centaurea paniculata [139]	115	-	0.1	0.1	-	-	0.1	-	-	1.7	-	-	tr.	-	-	1.1	-	0.2	-	0.3	-	-	-	-	-	-	17.5	-	-
Centaurea cariensis [150]	116	-	-	0.1	-	-	0.2	0.1	-	3.5	-	-	0.2	-	-	2.5	-	tr.	-	0.5	-	-	-	-	0.1	-	25.3	-	-
Centaurea aggregata [159]	117	0.3	0.1	0.3	-	-	-	-	-	0.3	-	-	-	-	-	1.0	-	-	-	0.8	-	-	-	-	-	-	37.8	-	-
Centaurea patula [158]	118	-	-	0.4	-	-	0.1	0.1	-	0.9	-	-	tr.	-	-	0.8	-	tr.	-	0.7	-	-	-	-	tr.	-	21.0	-	-
Centaurea pterocaula [157]	119	-	-	0.5	-	-	0.3	0.1	-	0.9	-	-	tr.	-	-	3.5	-	tr.	-	0.5	-	-	-	-	0.2	-	22.2	-	-
Centaurea depressa [159]	120	0.1	0.1	0.1	-	-	-	-	-	0.1	-	-	0.2	-	-	1.1	-	-	-	0.3	-	-	-	-	-	-	22.2	-	-
Centaurea pulchella [158]	121	-	-	0.2	-	-	0.3	0.3	-	0.5	-	-	tr.	-	-	1.3	-	0.2	-	0.3	-	-	-	-	0.1	-	19.4	-	-
Centaurea kotschyi [157]	122	-	-	0.1	-	-	0.1	0.1	-	0.4	-	-	0.1	-	-	1.3	-	tr.	-	0.2	-	-	-	-	0.1	-	12.0	-	-
Centaurea behen [159]	123	0.2	0.1	0.2	-	-	-	-	-	1.1	-	-	-	-	-	1.1	-	-	-	0.5	-	-	-	-	-	-	25.9	-	-
Centaurea cyanus [160]	124	-	0.2	0.1	-	-	0.1	-	-	-	-	-	-	-	-	0.9	-	0.2	-	0.4	-	-	-	-	-	-	15.4	-	-
Calendula officinalis [160]	125	-	0.3	0.3	-	-	0.2	0.1	-	1.7	-	-	-	-	-	9.9	-	-	-	0.2	-	-	-	-	-	-	27.1	-	-
Cichorium spinosum [120]	126	-	-	-	-	-	-	-	-	-	-	-	-	-	-	3.3	-	-	-	0.4	-	-	-	-	2.3	-	20.7	-	-
Cichorium intybus [129]	127	-	-	0.1	-	-	0.1	tr.	-	0.1	-	-	tr.	-	-	0.4	-	0.1	-	0.2	-	-	-	-	-	-	10.6	-	-
Cynara cornigera [120]	128	-	-	-	-	-	-	-	-	-	-	-	-	-	-	1.0	-	-	-	1.0	-	-	-	-	0.5	-	29.0	-	-
Cynara cardunculus [120]	129	-	-	-	-	-	-	-	-	-	-	-	-	-	-	-	-	-	-	-	-	-	-	-	0.7	-	34.7	-	-
Lactuca sativa [120]	130	-	-	-	-	-	-	-	-	-	-	-	-	-	-	1.9	-	0.3	-	0.2	-	-	-	-	-	-	13.0	-	-
Carthamus tinctorius [161]	131	-	-	-	-	-	37.7	-	-	-	-	-	-	-	-	-	-	-	-	-	-	-	-	-	-	-	16.0	-	-
Avicennia officinalis [135]	132	-	-	-	-	-	-	-	-	-	-	-	0.8	-	-	1.5	-	-	-	1.5	-	-	-	-	-	-	30.9	-	-
Acanthus ilicifolius [135]	133	-	-	-	-	-	-	-	-	38.6	-	-	1.5	-	-	1.5	-	-	-	1.2	-	-	-	-	-	-	32.0	-	-
Spathodea campanulata [134]	134	-	-	-	-	-	-	-	-	-	-	-	-	-	-	2.1	-	-	-	-	-	-	-	-	-	-	23.3	-	-
Aloysia citrodora [162]	135	-	-	-	-	-	-	-	-	-	-	-	-	-	-	1.9	-	-	-	-	-	-	-	-	-	-	32.0	-	-
Verbena officinalis [162]	136	-	-	-	-	-	-	-	-	-	-	-	-	-	-	0.9	-	-	-	-	-	-	-	-	-	-	21.1	-	-
Nepeta transcaucasica [163]	137	-	-	-	-	-	-	-	-	-	-	-	-	-	-	-	-	-	-	-	-	-	-	-	-	-	46.9	-	-
Lavandula X intermedia [164]	138	-	-	-	-	-	-	-	-	-	-	-	-	-	-	2.7	-	-	-	0.5	-	-	-	-	-	-	45.1	-	-
Thymbra capitata [165]	139	-	-	-	-	-	-	-	-	-	-	-	-	-	-	0.5	-	-	-	0.2	-	-	-	-	-	-	16.6	-	-
Thymus fallax [166]	140	-	-	-	-	-	-	-	-	0.1	-	-	-	-	-	0.4	-	-	-	0.2	-	-	-	-	-	-	21.9	-	-
Thymus kotschyanus var. glabrescens [166]	141	-	-	-	-	-	-	-	-	-	-	-	-	-	-	-	-	-	-	-	-	-	-	-	-	-	21.5	-	-
Thymus kotschyanus var. kotschyanus [166]	142	-	-	-	-	-	-	-	-	-	-	-	-	-	-	0.5	-	-	-	0.6	-	-	-	-	-	-	21.5	-	-
Thymus haussknechtii [166]	143	-	-	-	-	-	-	-	-	-	-	-	-	-	-	0.2	-	-	-	0.2	-	-	-	-	-	-	27.7	-	-
Thymus pubescens [166]	144	-	-	-	-	-	-	-	-	-	-	-	-	-	-	0.4	-	-	-	0.3	-	-	-	-	-	-	13.5	-	-
Vitex altissima [167]	145	-	-	-	-	-	-	-	-	0.1	-	-	-	-	-	0.1	-	-	-	-	-	-	-	-	-	-	37.2	-	-
Vitex negundo [167]	146	-	-	-	-	-	-	-	-	5.5	-	-	-	-	-	0.7	-	-	-	-	-	-	-	-	-	-	40.3	-	-
Vitex trifolia [167]	147	-	-	-	-	-	-	-	-	0.1	-	-	-	-	-	0.1	-	-	-	-	-	-	-	-	-	-	40.0	-	-
Scutellaria orientalis [163]	148	-	-	-	-	-	-	-	-	-	-	-	-	-	-	-	-	-	-	-	-	-	-	-	-	-	21.8	-	-
Satureja boissieri [166]	149	-	-	-	-	-	-	-	-	-	-	-	-	-	-	-	-	-	-	-	-	-	-	-	-	-	22.9	-	-
Satureja macrantha [163]	150	-	-	-	-	-	-	-	-	-	-	-	-	-	-	6.9	-	-	-	-	-	-	-	-	-	-	60.0	-	-
Satureja hortensis [164]	151	-	-	-	-	-	-	-	-	0.1	-	-	-	-	-	0.5	-	-	-	0.1	-	-	-	-	-	-	11.8	-	-
Prasium majus [120]	152	-	-	-	-	-	-	-	-	-	-	-	-	-	-	2.1	-	-	-	0.2	-	-	-	-	3.4	-	22.6	-	-
Origanum acutidens [166]	153	-	-	-	-	-	-	-	-	0.1	-	-	-	-	-	1.5	-	-	-	0.3	-	-	-	-	-	0.2	21.0	-	-
Origanum vulgare [166]	154	-	-	-	-	-	-	-	-	-	-	-	-	-	-	0.5	-	-	-	0.2	-	-	-	-	-	0.1	15.2	-	-
Ocimum basilicum [168]	155	-	-	-	-	-	-	-	-	0.2	-	-	-	-	-	0.4	-	-	-	0.3	-	-	-	-	-	-	36.6	-	-
Salvia euphratica [163]	156	-	-	-	-	-	-	-	-	-	-	-	-	-	-	-	-	-	-	-	-	-	-	-	-	-	37.3	-	-
Salvia officinalis [169]	157	-	-	-	-	-	-	-	-	-	-	-	-	-	-	-	-	-	-	-	-	-	-	-	-	-	12.4	-	-
Mentha cervina [170]	158	-	0.4	0.3	-	-	0.1	-	-	0.3	-	-	1.9	-	-	1.2	-	-	-	-	-	-	-	-	-	-	21.0	-	-
Mentha piperita [144]	159	-	-	-	-	-	0.5	-	-	1.8	-	-	-	-	-	0.7	-	-	-	0.7	-	-	-	-	-	-	23.3	-	-
Plantago lanceolata [122]	160	-	-	-	-	-	-	-	-	0.2	-	-	-	-	-	0.7	-	-	-	-	-	-	-	-	-	-	34.5	-	-
Antirrhinum sp. [122]	161	-	-	-	-	-	-	-	-	tr.	-	-	-	-	-	0.5	-	-	-	-	-	-	-	-	-	-	33.5	-	-
Olea europaea [171]	162	-	-	-	-	-	-	-	-	2.8	-	-	-	-	-	2.5	-	-	-	-	-	-	-	-	-	-	20.3	-	-
Solanum erianthum [127]	163	-	-	-	-	-	-	-	-	-	-	-	-	-	-	2.7	-	-	-	-	-	-	-	-	-	-	22.5	-	-
Solanum macrocarpon [125]	164	-	-	-	-	-	-	-	-	-	-	-	-	-	-	1.6	-	0.3	-	-	-	-	-	-	-	-	20.7	-	-
Solanum nigrum [120]	165	-	-	-	-	-	-	-	-	-	-	-	-	-	-	-	-	-	-	-	-	-	-	-	-	-	35.6	-	-
Nicotiana suaveolens [31]	166	-	-	-	-	-	-	-	-	-	-	-	-	-	-	-	-	-	-	-	-	-	-	-	-	-	17.1	-	-
Nicotiana arentsii [31]	167	-	-	-	-	-	-	-	-	-	-	-	-	-	-	-	-	-	-	-	-	-	-	-	-	-	14.3	-	-
Nicotiana wigandioides [31]	168	-	-	-	-	-	-	-	-	-	-	-	-	-	-	-	-	-	-	-	-	-	-	-	-	-	15.8	-	-
Nicotiana pauciflora [31]	169	-	-	-	-	-	-	-	-	-	-	-	-	-	-	-	-	-	-	-	-	-	-	-	-	-	14.0	-	-
Nicotiana setchellii [31]	170	-	-	-	-	-	-	-	-	-	-	-	-	-	-	-	-	-	-	-	-	-	-	-	-	-	15.7	-	-
Nicotiana raimondii [31]	171	-	-	-	-	-	-	-	-	-	-	-	-	-	-	-	-	-	-	-	-	-	-	-	-	-	15.0	-	-
Nicotiana rotundifolia [31]	172	-	-	-	-	-	-	-	-	-	-	-	-	-	-	-	-	-	-	-	-	-	-	-	-	-	16.3	-	-
Nicotiana maritima [31]	173	-	-	-	-	-	-	-	-	-	-	-	-	-	-	-	-	-	-	-	-	-	-	-	-	-	18.0	-	-
Nicotiana rosulata [31]	174	-	-	-	-	-	-	-	-	-	-	-	-	-	-	-	-	-	-	-	-	-	-	-	-	-	17.9	-	-
Nicotiana rosulata subsp. Ingulba [31]	175	-	-	-	-	-	-	-	-	-	-	-	-	-	-	-	-	-	-	-	-	-	-	-	-	-	18.2	-	-
Nicotiana occidentalis [31]	176	-	-	-	-	-	-	-	-	-	-	-	-	-	-	-	-	-	-	-	-	-	-	-	-	-	17.6	-	-
Nicotiana occidentalis subsp. Hesperis [31]	177	-	-	-	-	-	-	-	-	-	-	-	-	-	-	-	-	-	-	-	-	-	-	-	-	-	16.7	-	-
Nicotiana goodspeedii [31]	178	-	-	-	-	-	-	-	-	-	-	-	-	-	-	-	-	-	-	-	-	-	-	-	-	-	17.6	-	-
Nicotiana africana [31]	179	-	-	-	-	-	-	-	-	-	-	-	-	-	-	-	-	-	-	-	-	-	-	-	-	-	17.5	-	-
Nicotiana corymbosa [31]	180	-	-	-	-	-	-	-	-	-	-	-	-	-	-	-	-	-	-	-	-	-	-	-	-	-	15.5	-	-
Nicotiana solanifolia [31]	181	-	-	-	-	-	-	-	-	-	-	-	-	-	-	-	-	-	-	-	-	-	-	-	-	-	15.6	-	-
Nicotiana undulata [31]	182	-	-	-	-	-	-	-	-	-	-	-	-	-	-	-	-	-	-	-	-	-	-	-	-	-	13.5	-	-
Nicotiana umbratica [31]	183	-	-	-	-	-	-	-	-	-	-	-	-	-	-	-	-	-	-	-	-	-	-	-	-	-	16.4	-	-
Nicotiana trigonophylla [31]	184	-	-	-	-	-	-	-	-	-	-	-	-	-	-	-	-	-	-	-	-	-	-	-	-	-	17.3	-	-
Nicotiana stocktonii [31]	185	-	-	-	-	-	-	-	-	-	-	-	-	-	-	-	-	-	-	-	-	-	-	-	-	-	18.3	-	-

Nicotiana simulans [31]	186	17.9 · 5.7 · 4.0 · 3.8 · 1.4 · 10.9 · 56.3
Nicotiana noctiflora [31]	187	16.4 · 5.7 · 6.2 · 2.9 · 1.7 · 13.1 · 54.0
Nicotiana miersii [31]	188	14.8 · 6.7 · 5.6 · 2.2 · 1.0 · 14.1 · 55.6
Nicotiana megalosiphon [31]	189	18.6 · 5.8 · 3.6 · 3.2 · 1.5 · 10.2 · 57.1
Nicotiana longiflora [31]	190	17.2 · 4.4 · 6.3 · 3.3 · 1.6 · 13.4 · 53.8
Nicotiana langsdorffii [31]	191	15.2 · 6.4 · 5.6 · 2.8 · 0.8 · 10.1 · 59.1
Nicotiana knightiana [31]	192	16.1 · 6.9 · 6.5 · 2.4 · 2.8 · 8.4 · 56.9
Nicotiana gossei [31]	193	18.5 · 4.8 · 4.2 · 3.6 · 1.6 · 10.2 · 57.1
Nicotiana exigua [31]	194	15.7 · 6.7 · 7.7 · 1.9 · 1.7 · 11.4 · 54.9
Nicotiana bonariensis [31]	195	15.5 · 4.3 · 5.6 · 2.6 · 0.7 · 11.7 · 59.6
Nicotiana amplexicaulis [31]	196	16.8 · 6.3 · 4.4 · 3.0 · 1.7 · 5.0 · 62.8
Nicotiana cavicola [31]	197	15.8 · 5.8 · 7.7 · 3.8 · 1.3 · 9.0 · 56.6
Nicotiana clevelandii [31]	198	17.5 · 7.3 · 5.8 · 3.0 · 1.2 · 13.0 · 52.2
Nicotiana repanda [31]	199	16.7 · 6.4 · 4.2 · 2.7 · 0.8 · 15.1 · 54.1
Nicotiana paniculata [31]	200	17.1 · 7.0 · 6.0 · 2.7 · 2.8 · 8.0 · 56.4
Nicotiana excelsior [31]	201	17.5 · 7.3 · 7.1 · 3.5 · 1.4 · 6.3 · 56.9
Nicotiana tomentosa [31]	202	17.2 · 4.9 · 3.8 · 3.6 · 2 · 10.7 · 57.8
Nicotiana kawakamii [31]	203	16.0 · 7.1 · 5.5 · 5.4 · 1.7 · 11.6 · 55.7
Nicotiana nudicaulis [31]	204	17.0 · 7.2 · 7.2 · 2.2 · 1.2 · 12.7 · 52.5
Nicotiana attenuata [31]	205	14.9 · 6.3 · 5.0 · 2.7 · 1.1 · 13.7 · 56.3
Nicotiana glutinosa [31]	206	16.1 · 7.4 · 6.3 · 1.8 · 0.9 · 8.9 · 58.6
Nicotiana benthamiana [31]	207	16.4 · 6.8 · 4.9 · 2.8 · 2.8 · 5.7 · 60.6
Nicotiana tomentosiformis [31]	208	14.9 · 4.4 · 5.0 · 2.8 · 1.9 · 10.7 · 60.3
Nicotiana tabacum [172]	209	2.5 · 2.6 · 1.3 · 2.1 · 0.5 · 19.5 · 3.8 · 1.5 · 6.1 · 2.6 · 1.6 · 12.5 · 0.3 · 42.5 · 0.6
Nicotiana sylvestris [31]	210	14.8 · 6.3 · 6.1 · 3.0 · 2.1 · 10.6 · 57.1
Nicotiana rustica [31]	211	16.8 · 6.8 · 4.6 · 2.2 · 1.5 · 8.6 · 59.5
Nicotiana plumbaginifolia [31]	212	15.4 · 6.6 · 6.7 · 2.3 · 2.4 · 15.2 · 51.4
Nicotiana otophora [31]	213	14.8 · 2.7 · 5.2 · 2.6 · 1.3 · 10.0 · 63.4
Nicotiana glauca [31]	214	14.8 · 6.8 · 7.4 · 1.7 · 1.8 · 17.3 · 50.2
Nicotiana debneyi [31]	215	17.0 · 7.0 · 5.2 · 3.0 · 1.0 · 8.7 · 58.1
Nicotiana quadrivalvis var. bigelovii [31]	216	15.4 · 6.0 · 4.8 · 1.8 · 2.0 · 11.4 · 58.6
Nicotiana alata [31]	217	15.4 · 6.1 · 5.2 · 3.0 · 1.0 · 12.2 · 57.1
Nicotiana acuminata [31]	218	15.2 · 6.8 · 4.2 · 2.5 · 1.1 · 13.5 · 56.7
Capsicum annuum [173]	219	2.4 · 7.2 · 18.1 · 0.8 · 4.73 · 8.5 · 27.2 · 29.9
Hedera helix [122]	220	4.1 · 3.6 · 15.8 · 4.03 · 20.0 · 20.1 · 18.3 · 2.6
Anethum foeniculum [120]	221	3.1 · 0.3 · 0.3 · 24.0 · 1.5 · 0.3 · 1.6 · 1.2 · 1.3 · 29.0 · 0.1 · 32.7 · 0.8 · 1.1 · 0.8 · 1.5 · 0.3
Helosciadium nodiflorum [129]	222	0.7 · tr. · 0.3 · 0.7 · 0.9 · 0.4 · 16.3 · 1.1 · 1.8 · 3.3 · 24.6 · 43.5 · 0.3 · 2.6 · 1.2 · 2.1
Centella asiatica [174]	223	13.7 · 0.5 · 55.7 · 8.6 · 17.5 · 4.0
Crithmum maritimum [175]	224	26.0 · 1.9 · 1.7 · 7.8 · 3.2 · 4.2 · 30.1 · 25.7
Coriandrum sativum [176]	225	5.7 · 0.6 · 0.1 · 5.5 · 0.2 · 0.9 · 5.2 · 14.7 · 2.6
Apium graveolens [120]	226	1.4 · 0.5 · 22.1 · 1.2 · 0.5 · 4.5 · 5.6 · 24.6 · 27.1 · 3.0 · 6.4 · 6.0 · 1.6 · 1.4
Petroselinum crispum [120]	227	2.9 · 0.2 · 0.4 · 21.8 · 1.3 · 0.3 · 1.4 · 8.9 · 10.8 · 21.9 · 24.3 · 1.2 · 3.1 · 0.3 · 1.1
Daucus carota [120]	228	2.4 · 0.2 · 0.2 · 21.1 · 1.4 · 0.2 · 1.8 · 12.1 · 25.7 · 28.1 · 2.9 · 1.5 · 0.3 · 1.9
Salix sp. [122]	229	3.4 · 4.5 · 15.8 · 12.9 · 22.4 · 9.6 · 21.7 · 2.9
Ceriops decandra [135]	230	24.9 · 0.8 · 7.2 · 0.6 · 45.6 · 1.0 · 0.1 · 9.5 · 4.2 · 3.7 · 0.4 · tr. · 0.4
Bruguiera cylindrica [135]	231	12.2 · 0.6 · 8.6 · 1.3 · 56.3 · 1.3 · 0.5 · 9.6 · 3.0 · tr. · 2.3 · 0.3 · tr. · 0.6
Rhizophora apiculata [135]	232	19.3 · 2.4 · 0.3 · 55.0 · 0.9 · 0.1 · 5.0 · 7.0 · 8.3 · tr. · tr. · 0.2
Rhizophora mucronata [135]	233	12.5 · 3.1 · 0.7 · 59.1 · 0.9 · 0.6 · 7.7 · 4.3 · 8.8 · tr. · tr. · 0.3
Cnidoscolus aconitifolius [177]	234	0.2 · 0.2 · 0.2 · 0.5 · 0.1 · 22.7 · 0.4 · 0.8 · 4.8 · 8.3 · 1.0 · 8.9 · 49.7 · 0.5 · 0.1 · 0.1 · 0.3 · 0.3 · 0.4 · 0.2
Croton zambesicus [178]	235	1.0 · 1.4 · 32.0 · 3.0 · 4.8 · 33.3 · 18.3 · 2.9 · 0.6 · 1.4 · 0.7 · 0.6
Excoecaria agallocha [179]	236	18.1 · 0.8 · 3.6 · 2.7 · 56.0 · 1.0 · 2.8 · 1.7 · 3.1 · tr. · 7.0 · 0.1 · 0.8
Euphorbia lathyris [180]	237	3.1 · 12.5 · 1.3 · 1.9 · 12.5 · 38.3
Euphorbia lagascae [180]	238	3.2 · 9.5 · 1.1 · 1.0 · 9.3 · 38.0 · 0.4
Linum usitatissimum [181]	239	0.1 · 11.0 · 1.1 · 21.6 · 2.3 · 14.2 · 3.4 · 42.9 · 0.9
Melilotus siculus [143]	240	0.5 · 30.0 · 3.0 · 58.0
Senna sophera [182]	241	0.9 · 1.5 · 1.4 · 1.2 · 0.6 · 1.0 · 0.7 · 1.0 · 0.4 · 1.2 · 15.9 · 4.0 · 4.6 · 9.5 · 8.8 · 38.7 · 1.9 · 0.7 · 0.4 · 3.3 · 0.7 · 0.7
Senna tora [183]	242	0.1 · 0.6 · 0.1 · 1.0 · 0.8 · 18.6 · 1.5 · 6.3 · 11.2 · 13.2 · 0.1 · 16.1 · 2.1 · 0.7 · 1.3 · 0.8 · 1.6 · 0.4 · 1.3 · 0.9 · 3.5
Senna siamea [134]	243	2.9 · 54.4 · 13.9 · 19.3 · 15.8 · 5.0
Delonix regia [134]	244	4.7 · 4.8 · 36.0 · 9.7 · 18.2 · 14.7 · 7.7 · 4.8
Trifolium angustifolium [139]	245	2.0 · 0.8 · 0.2 · 2.3 · tr. · 4.8 · 0.2 · 0.3 · 15.4 · 0.3 · 0.4 · 3.2 · 6.1 · 20.2 · 34.7 · 2.6 · 0.2 · 0.1 · 0.4 · 2.4 · 0.2 · 3.2
Trifolium alexandrinum [143]	246	26.3 · 18.5 · 3.6 · 8.7 · 7.6 · 29.9
Trifolium pratense [122]	247	tr. · 1.2 · 38.3 · 2.3 · 10.5 · 5.0 · 41.4 · tr.
Cassia fistula [134]	248	2.1 · 36.5 · 15.2 · 41.3 · 2.8
Ceratonia siliqua [132]	249	0.2 · 1.2 · 0.1 · 4.7 · 0.3 · 8.3 · 0.3 · 0.3 · 0.5 · 42.0 · 0.1 · 1.6 · 0.1 · 9.9 · 4.0 · 2.6 · 18.8 · 4.2 · tr. · 0.1 · 0.8
Vigna unguiculata [125]	250	3.2 · 0.4 · 29.2 · 0.4 · 4.6 · 1.9 · 7.2 · 50.4 · 0.9 · 1.3 · 0.5
Vicia faba [120]	251	0.3 · 0.3 · 0.5 · 17.9 · 2.8 · 5.3 · 52.1 · 14.2 · 4.8 · 3.4 · 1.0 · 0.3
Onobrychis viciifolia [122]	252	tr. · 1.8 · 35.6 · 4.1 · 9.3 · 14.3 · 32.8 · tr.
Lathyrus ochrus [120]	253	2.2 · 0.3 · 0.2 · 17.0 · 0.7 · 0.5 · 1.1 · 7.2 · 2.5 · 13.9 · 0.2 · 50.4 · 1.1 · 0.6 · 1.7 · 0.4
Moringa oleifera [184]	254	0.6 · tr. · 0.1 · 0.3 · 0.9 · 18.4 · 2.5 · 4.3 · 3.3 · 11.0 · 54.3 · 1.1 · 0.7 · 1.0 · 1.6
Arabidopsis halleri [185]	255	tr. · 0.4 · 0.2 · 19.5 · 0.4 · 0.5 · 0.8 · 8.5 · 1.5 · 5.0 · 1.2 · 0.7 · 19.8 · 40.1 · 0.3 · 0.1 · 0.2 · 0.1 · 0.1 · tr. · 0.3 · 0.3 · tr. · tr.
Arabidopsis lyrata [185]	256	0.1 · 1.3 · 0.9 · 29.4 · 1.5 · 1.8 · 0.2 · 5.0 · 4.2 · 4.1 · 1.1 · 0.3 · 20.0 · 28.2 · 0.3 · 0.2 · 0.1 · 0.4 · 0.9
Sinapis arvensis [143]	257	0.9 · 0.2 · 20.7 · 2.8 · 14.9 · 10.1 · 3.6 · 6.3 · 28.6 · 1.6
Eruca vesicaria subsp. Sativa [120]	258	2.9 · 0.3 · 4.7 · 25.9 · 1.1 · 0.4 · 0.1 · 2.9 · 2.5 · 10.3 · 0.4 · 38.9 · 2.4 · 2.1 · 0.2 · 4.6 · 0.4
Brassica napus [186]	259	0.2 · 0.6 · 23.5 · 1.1 · 0.8 · 2.1 · 7.3 · 55.0 · 0.3 · 0.3 · 0.6 · 0.5 · 2.8
Cucurbita pepo [187]	260	0.5 · 0.4 · 30.1 · 4.0 · 7.2 · 3.6 · 5.1 · 41.9 · 2.1 · 1.8 · 1.9
Cucurbita pepo subsp. pepo [187]	261	2.3 · 0.6 · 18.0 · 0.7 · 4.9 · 5.7 · 16.2 · 33.3 · 2.4 · 0.1 · 5.6 · 3.9 · 3.7
Cucurbita pepo var. melopepo [187]	262	3.0 · 0.7 · 16.6 · 1.1 · 3.5 · 4.7 · 27.5 · 32.5 · 3.0 · 2.3 · 1.9
Bryonia cretica [120]	263	0.9 · 1.1 · 26.4 · 1.4 · 0.4 · 0.2 · 1.0 · 4.8 · 33.6 · 0.2 · 25.4 · 0.3 · 1.5 · 0.6 · 0.6 · 2.5 · 0.2
Bryonia dioica [129]	264	tr. · 0.1 · 0.1 · tr. · 0.1 · 0.3 · 0.1 · 0.2 · 17.0 · 0.1 · 0.4 · 2.7 · 1.2 · 6.5 · 67.8 · 0.5 · 0.2 · 1.0 · 0.3 · 1.6
Montia fontana [129]	265	0.1 · 0.1 · 0.2 · 0.6 · 0.3 · 0.4 · 11.3 · 0.3 · 0.2 · 1.0 · 6.1 · 18.2 · 0.4 · 47.7 · 9.5 · 0.1 · 0.1 · 0.7 · 1.5 · 0.7 · 0.1 · 0.6
Sesuvium portulacastrum [188]	266	1.7 · 0.5 · 6.9 · 0.1 · 38.2 · 0.1 · 21.2 · 10.6 · 14.2 · 0.3 · tr. · 2.4
Amaranthus cruentus [125]	267	1.9 · 22.4 · 0.3 · 2.8 · 3.2 · 0.3 · 17.2 · 50.1 · 0.4 · 0.7 · 0.5
Portulaca oleracea [189]	268	2.0 · 11.7 · 2.7 · 5.5 · 12.1 · 62.5 · 1.2 · 1.6 · 0.8
Silene vulgaris [120]	269	1.8 · 0.3 · 21.7 · 1.0 · 0.3 · 1.0 · 1.4 · 1.2 · 26.8 · 0.2 · 39.6 · 0.4 · 1.6 · 0.3 · 2.4
Rumex pulcher [129]	270	tr. · 0.1 · 0.1 · tr. · 0.2 · tr. · 0.4 · 0.1 · 0.2 · 9.3 · 0.2 · 0.3 · 1.7 · 4.2 · 17.0 · 63.0 · 0.4 · 0.1 · 0.2 · 0.2 · 0.1 · 0.9 · 0.1 · 0.1 · 0.9 · 0.1
Rumex papillaris [129]	271	0.1 · tr. · tr. · 0.1 · 0.5 · 2.8 · 0.7 · 11.2 · 0.2 · 0.2 · 0.8 · 5.8 · 22.8 · 51.8 · 0.3 · 0.2 · 0.3 · 0.1 · 0.8 · 0.1 · 0.1 · 0.1 · 1.2 · 0.1
Rumex patientia [190]	272	7.6 · 0.1 · 0.2 · 0.3 · 0.2 · 11.3 · 0.7 · 0.1 · 0.5 · 1.2 · 0.3 · 4.3 · 13.6 · 0.4 · 11.6 · 43.2 · 0.3 · 0.1 · 0.3 · 0.4 · 0.1 · 0.3 · 0.3 · 1.0 · 1.0 · 0.5
Rumex obtusifolius [120]	273	2.3 · 0.3 · 0.1 · 20.0 · 0.8 · 0.4 · 0.3 · 3.1 · 3.3 · 21.9 · 0.3 · 42.3 · 2.8 · 1.3 · 0.8 · 0.8 · 0.4 · 0.5
Salicornia europaea [16]	274	1.5 · 19.4 · 1.4 · 1.5 · 3.3 · 0.6 · 22.5 · 41.4 · 0.5 · 4.8 · 1.2 · 0.8 · 1.3
Salicornia brachiata [191]	275	61.9 · 1.3 · 1.2 · 0.5 · 19.1 · tr. · 0.2 · tr. · 10.6 · tr. · 4.3 · tr. · tr. · tr.
Beta vulgaris [120]	276	1.0 · 2.4 · 23.6 · 0.3 · 0.3 · 1.0 · 4.7 · 25.0 · 38.9 · 0.3 · 3.4 · 0.7 · 0.3 · 0.3 · 0.7
Suaeda monoica [191]	277	10.8 · 0.2 · 2.6 · 1.4 · 43.1 · 0.2 · tr. · 5.3 · 22.2 · 8.4 · 0.6 · tr. · 4.2
Suaeda altissima [192]	278	0.1 · 0.5 · 0.2 · 23.1 · 0.1 · 1.0 · 0.1 · 0.1 · 2.2 · 7.0 · 0.5 · 22.1 · 0.1 · 39.0 · 1.0 · 0.3 · 0.1 · 0.1 · 0.1 · 0.7 · 0.2 · 0.8 · 0.3 · 0.3
Suaeda maritima [191]	279	23.9 · 0.4 · 1.6 · 0.9 · 41.9 · 0.3 · 1.1 · 3.9 · 14.3 · 6.2 · 0.7 · 0.1 · 3.2
Spinacia oleracea [120]	280	1.4 · 0.3 · 16.8 · 1.1 · 0.4 · 0.2 · 1.4 · 12.7 · 14.4 · 0.2 · 40.9 · 1.3 · 4.0 · 0.8 · 1.7 · 0.2 · 1.6 · 4.2
Chenopodium album [193]	281	tr. · 0.2 · 0.1 · 19.8 · 0.1 · 1.9 · 1.6 · 0.8 · 2.9 · 0.3 · 14.3 · 57.4 · 0.2 · 0.3 · 0.1
Hippophae rhamnoides [137]	282	1.6 · 1.3 · 0.5 · 1.4 · 29.3 · 47.8 · 1.0 · 6.5 · 10.6 · tr. · tr. · tr. · tr.
Rubus ulmifolius [139]	283	0.3 · 0.1 · 0.1 · 0.6 · tr. · 1.1 · 0.1 · 0.2 · 12.0 · 0.2 · 0.3 · 3.1 · 4.3 · 16.0 · 39.6 · 6.1 · tr. · 0.1 · 0.5 · 8.7 · 0.3 · 6.2
Rosa elliptica subsp. inodora [194]	284	0.1 · tr. · 3.2 · 0.1 · 0.1 · 0.1 · 2.4 · 0.1 · 18.2 · 0.5 · 50.3 · 22.8 · 0.8 · 0.3 · 0.1 · 0.2 · tr.
Rosa dumalis [194]	285	tr. · tr. · 2.9 · 0.1 · 0.1 · 0.1 · 1.8 · 0.1 · 14.4 · 0.5 · 48.4 · 29.5 · 0.7 · 0.4 · 0.1 · 0.1 · tr.
Rosa villosa [194]	286	tr. · tr. · 3.1 · 0.1 · 0.1 · 0.1 · 1.7 · 0.1 · 18.4 · 0.5 · 44.4 · 29.5 · 0.8 · 0.6 · 0.2
Rosa subcanina [194]	287	tr. · tr. · 3.1 · tr. · 0.1 · 0.1 · 1.9 · 0.1 · 13.9 · 0.5 · 49.8 · 28.7 · 0.7 · 0.4 · 0.1 · 0.2 · tr.
Rosa micrantha [195]	288	0.1 · 0.4 · 0.3 · 0.9 · 1.5 · 0.2 · 1 · 11.3 · 0.7 · 6.7 · 1.8 · 21.2 · 32.3 · 3.7 · 0.6 · 0.3 · 4.4 · 9.3 · 3.4
Rosa rugosa [194]	289	tr. · tr. · 2.8 · tr. · 0.2 · 0.1 · 1.0 · 0.1 · 14.4 · 0.7 · 50.3 · 27.9 · 0.7 · 0.7 · 0.2 · 0.3 · tr.
Rosa rubiginosa [194]	290	0.1 · tr. · 2.9 · tr. · tr. · 0.1 · 1.6 · tr. · 14.2 · 0.6 · 47.2 · 31.4 · 0.7 · 0.4 · 0.1 · 0.2 · 0.1
Rosa canina [194]	291	tr. · tr. · 3.5 · tr. · 0.1 · 0.1 · 2.5 · tr. · 20.3 · 0.5 · 51.7 · 19.1 · 0.9 · 0.3 · 0.1 · 0.2 · 0.1
Rosa majalis [144]	292	1.2 · 0.4 · 26.1 · 2.2 · 0.9 · 2.9 · 3.8 · 20.3 · 39.0 · 0.7 · 0.6 · 2.2 · 0.1
Malus orientalis [196]	293	1.6 · 0.4 · 19.5 · 0.4 · 15.2 · 13.7 · 0.3 · 24.3 · 0.2 · 8.8 · 7.8 · 0.4 · 0.5 · 5.0 · 1.9
Malus domestica [197]	294	0.4 · 0.1 · 1.4 · 0.4 · 20.7 · 0.3 · 0.4 · 0.1 · 10.6 · 6.9 · 15.9 · 0.7 · 3.1 · 0.1 · 31.0 · 0.4 · 6.2 · 1.0 · 0.1 · tr. · 0.2
Mespilus germanica [47]	295	0.8 · 0.1 · 1.3 · 0.5 · 24.0 · 1.3 · 0.3 · 0.2 · 7.9 · tr. · 6.9 · 0.7 · 22.5 · 0.3 · 27.4 · 2.3 · 0.1 · 0.1 · 2.0 · tr. · 1.3
Cydonia oblonga [198]	296	0.7 · 0.1 · 0.7 · 0.4 · 24.7 · 0.6 · 0.5 · tr. · 0.1 · 3.9 · 0.2 · 9.3 · 1.7 · tr. · 26.6 · 0.3 · 27.8 · 1.0 · 0.5 · 0.1 · 0.4 · tr. · tr.
Pyrus communis [17]	297	0.9 · 1.3 · 0.5 · 32.0 · 0.3 · 1.2 · 0.1 · 0.5 · 7.1 · 3.2 · 7.9 · 41.8 · 1.2 · 0.2 · 1.2 · 0.6
Pyrus caucasica [17]	298	0.6 · 1.4 · 0.3 · 30.4 · 0.1 · 0.9 · 0.1 · 0.6 · 5.9 · 7.8 · 8.1 · 31.8 · 7.4 · 0.3 · 3.1 · 1.2
Eriobotrya japonica [132]	299	0.1 · 0.5 · tr. · 3.8 · 0.1 · 2.8 · 0.1 · 0.2 · 0.5 · 24.3 · 0.4 · 0.9 · 0.4 · 3.5 · 8.9 · 18.9 · 0.8 · 32.5 · 0.3 · 0.3 · 0.2 · 0.6
Prunus spinosa [199]	300	0.2 · 0.3 · 0.2 · tr. · 1.2 · tr. · 6.7 · tr. · 0.2 · tr. · 19.9 · 1.2 · 0.2 · 0.2 · 9.4 · 26.0 · 3.0 · tr. · 2.6 · 2.7 · 1.1 · tr. · tr. · tr. · 0.1 · tr. · tr. · 1.1 · 5.1 · tr. · tr. · tr. · 0.4 · 0.6 · tr.
Prunus dulcis [200]	301	0.2 · 0.4 · 0.5 · 23.3 · 3.9 · 10.4 · 33.5 · 22.6 · 1.7 · 2.3 · 0.6
Urtica dioica [201]	302	1.3 · 3.0 · 0.5 · 20.2 · 1.0 · 0.3 · 0.5 · 3.3 · 3.2 · 18.2 · 22.6 · 6.9 · 2.8 · 0.9 · 0.5 · 5.9 · 1.3 · 1.1 · 4.3 · 1.7
Morus nigra [202]	303	2.2 · 0.5 · 26.0 · 0.3 · 1.9 · 5.3 · 2.3 · 16.1 · 37.6 · 4.1 · 1.2 · 1.9 · 0.6
Morus alba [132]	304	1.6 · 2.1 · 1.7 · 8.9 · 0.9 · 1.5 · 1.3 · 0.6 · 0.6 · 14.2 · 4.7 · 0.3 · 0.5 · 3.1 · 5.4 · 23.4 · 2.4 · 26.8 · 0.2 · tr. · tr. · tr. · tr. · tr.
Ficus carica [132]	305	0.1 · 0.1 · tr. · 1.9 · tr. · 3.1 · 0.1 · 1.0 · 0.5 · 25.2 · 0.4 · 3.1 · 0.1 · 8.4 · 4.2 · 10.6 · 13.7 · 25.6 · 0.1 · tr. · 0.9 · 1.0 · tr. · tr.
Humulus lupulus [129]	306	tr. · 0.1 · 0.1 · 0.5 · 0.1 · 0.1 · 19.5 · 0.5 · 0.3 · 1.6 · 1.9 · 29.7 · 0.4 · 38.2 · 2.3 · 0.1 · 0.3 · 0.4 · 2.9 · 0.9
Persea americana [132]	307	0.2 · 0.2 · 0.6 · 3.6 · 1.1 · 4.3 · 0.5 · 0.2 · 3.9 · 30.9 · 0.2 · 1.0 · tr. · 3.1 · 4.4 · 16.2 · 0.6 · 23.9 · 0.1 · tr. · 2.6 · 1.4 · 0.1 · 1.0
Pistacia lentiscus [203]	308	2.5 · 0.8 · 0.9 · 0.9 · 6.4 · tr. · 0.7 · 7.5 · 0.5 · 0.9 · 3.7 · 14.7 · 48.9 · 0.8 · tr. · 0.7 · tr. · tr. · 0.9 · tr.
Azadirachta indica [204]	309	0.3 · 0.1 · 0.3 · 1.0 · 0.5 · 1.9 · 0.8 · 31.8 · 0.3 · 2.9 · 9.7 · 18.6 · 12.6 · 1.3 · 0.5 · 7.4 · 2.1 · 5.7 · 0.5 · 1.7
Citrus limon [132]	310	tr. · 0.3 · 0.2 · 5.1 · 0.3 · 9.9 · 0.1 · 0.4 · 0.5 · 37.1 · 0.3 · 1.8 · 0.6 · 6.7 · 5.1 · 13.3 · 1.7 · 14.6 · 0.3 · 0.4 · 0.2 · 1.3
Ephedra chilensis [205]	311	0.6 · 1.0 · 15.2 · 0.9 · 0.2 · 0.3 · 1.2 · 6.2 · 1 · 29.8 · 0.2 · 29.8 · 0.6 · 0.9 · 0.4 · 0.8 · 0.7 · 3.3 · 2.3 · 2.5
Ephedra equisetina [205]	312	0.4 · 0.4 · 0.9 · 15.4 · 1.0 · 0.3 · 0.3 · 1.5 · 4.3 · 5.4 · 16.6 · 1.3 · 37.6 · 1.2 · 1.8 · 0.6 · 1 · 1.1 · 3.1 · 3.5 · 2.0
Ephedra fragilis [205]	313	0.5 · 0.4 · 1.4 · 18.0 · 1.5 · 0.6 · 2.2 · 7.5 · 1 · 16.7 · 43.2 · 0.3 · 0.9 · 1 · 1.2 · 1.2 · 1.8
Ephedra gerardiana [205]	314	0.9 · 0.6 · 17.9 · 0.7 · 0.5 · 0.4 · 2.3 · 7.0 · tr. · 19.3 · tr. · 37.5 · 0.3 · 1.4 · 0.2 · 0.2 · 0.9 · 4.2 · 2.5 · 3.1
Ephedra distachya [205]	315	0.5 · 0.7 · 0.4 · 20.1 · 1.2 · 0.6 · 3.3 · 7.6 · 27.7 · tr. · 28.4 · 1.7 · tr. · 1.2 · 3.4 · 0.5 · 2.1
Gnetum gnemon [205]	316	1.1 · 0.3 · 1.0 · 17.0 · 0.9 · 0.4 · 0.5 · 3.2 · 8.2 · 1.1 · 2.8 · 4.2 · 29.9 · 3.5 · 10.9 · 0.7 · 1.6 · 12.9
Welwitschia mirabilis [205]	317	0.4 · 1.0 · 1.1 · 23.9 · 1.7 · 1.2 · 0.5 · 0.4 · 1.9 · 11.4 · 7.6 · 45.6 · 0.9 · 0.9 · 0.3 · 0.4
Ginkgo biloba [206]	318	1.0 · 1.9 · 20.5 · 5.3 · 1.1 · 6.5 · 5.3 · 45.1 · 0.7 · 1.8 · 2.0 · 0.5 · 5 · 3.5
Ceratozamia robusta [205]	319	0.4 · 2.0 · 2.1 · 18.2 · 2.3 · 0.9 · 1.5 · 1.2 · 1.9 · 9.9 · 0.5 · 18.9 · 0.2 · 34.2 · 0.2 · 0.5 · 0.2 · 2.5 · 1.2 · 1.1
Lepidozamia hopei [205]	320	0.5 · 0.9 · 1.5 · 17.6 · tr. · 1.5 · 1.1 · 0.2 · 3.7 · 8.3 · 12.8 · 0.2 · 40.1 · 0.4 · 0.2 · 0.6 · 0.4 · 2.8 · 4.6 · 2.0
Macrozamia riedlei [207]	321	0.3 · 0.2 · 0.9 · 28.2 · 6.5 · 29.3 · 15.2 · 12.2 · 0.9
Macrozamia moorei [205]	322	0.3 · 0.5 · 1.2 · 18.3 · 0.6 · 0.3 · 1.7 · 0.6 · 3.1 · 7.2 · 19.9 · 0.2 · 36.9 · 0.4 · 0.4 · 0.4 · 2.0 · 4.8 · 1.2
Microcycas calocoma [205]	323	0.8 · 2.4 · tr. · 17.7 · 0.5 · 1.6 · 0.7 · tr. · 3.5 · 10.4 · 0.6 · 4.0 · tr. · 47.9 · 0.4 · 0.5 · tr. · 0.4 · 0.4 · 1.6 · 3.1 · 2.6 · 0.6
Encephalartos lebomboensis [205]	324	0.8 · 1.1 · 1.7 · 21.3 · 0.5 · 1.6 · 0.9 · 4.9 · 12.6 · 9.6 · 1.1 · 24.9 · 0.5 · 0.8 · 0.4 · 0.9 · 3.9 · 4.9 · 7.9
Dioon edule [205]	325	0.6 · 2.2 · 1.7 · 18.3 · 1.1 · 0.5 · 1.8 · 0.5 · 2.7 · 8.1 · tr. · 17.0 · 0.5 · 33.1 · 1.3 · 1.2 · 0.5 · 0.4 · 1.4 · 5.0 · 2.8
Zamia furfuracea [208]	326	0.2 · 0.5 · 1.6 · 15.7 · 2.0 · 0.7 · 1.8 · 0.2 · 1.9 · 7.7 · 5.8 · 0.2 · 41.0 · 0.7 · 5.9 · 0.4 · 1.3 · 0.4 · 1.7 · 6.0 · 4.4
Stangeria eriopus [205]	327	1.9 · 2.2 · 2.1 · 23.4 · 2.3 · 0.4 · 1.9 · 4.6 · 9.4 · 10.5 · 32.4 · 1.7 · 1.3 · 0.3 · 2.3 · 1.9 · 1.1
Bowenia spectabilis [205]	328	2.2 · 1.1 · 1.7 · 15.1 · 1.6 · 1.7 · 0.8 · 1.7 · 4.0 · 7.8 · 16.3 · 36.5 · 4.4 · 1.8 · 1.5 · 1.8
Cycas armstrongii [205]	329	1.4 · 1.5 · 18.5 · 2.7 · 1.3 · 2.3 · 1.0 · 5.0 · 7.3 · 13.2 · 0.6 · 43.0 · 0.2 · 1.1 · 0.4 · 0.5
Cycas siamensis [205]	330	1.5 · 1.4 · 20.3 · 2.9 · 0.4 · 3.6 · 1.2 · 2.6 · 10.1 · 0.5 · 14.4 · 0.3 · 38.3 · 0.3 · 0.2 · 0.3 · tr. · 0.6 · 0.5 · 0.5
Cycas revoluta [209]	331	0.6 · 2.7 · 0.4 · 1.3 · 22.9 · 0.2 · 1.4 · 1.7 · 1.3 · 1.0 · 0.2 · 3.8 · 5.7 · 1.1 · 0.4 · 1.5 · 0.1 · 27.1 · 0.5 · 23.9 · 1.1 · 0.1 · 0.8 · 0.3
Pectinopitys ferruginea [205]	332	0.5 · 1.0 · 1.1 · 23.4 · 1.3 · 1.0 · 1.4 · 1.8 · 6.6 · 19.1 · 33.5 · 1.2 · 1.6 · 0.6 · 2.5 · 2.5 · 0.9
Podocarpus salignus [205]	333	2.3 · 5.8 · 1.1 · 19.5 · 1.4 · 0.6 · 0.8 · 0.2 · 1.1 · 4.6 · 0.2 · 14.2 · 39.6 · 0.4 · 0.2 · 0.3 · 0.6 · 3.5 · 2.9 · 0.3 · 2.5
Podocarpus nivalis [205]	334	0.5 · 0.4 · 17.8 · 0.7 · 0.5 · tr. · 16.0 · 1.4 · 2.4 · 13.0 · 26.3 · 5.4 · 2.8 · 0.8 · 2.5 · 1.6 · 3.6 · 4.3
Podocarpus macrophyllus [205]	335	1.8 · 2.9 · 23.8 · 1.4 · 0.2 · 0.6 · 1.0 · 1.6 · 3.4 · 11.3 · 32.7 · 3.3 · 10.5 · 4.1
Podocarpus macrophyllus var. maki [205]	336	0.9 · 4.3 · 0.8 · 21.8 · 1.6 · 0.5 · 0.9 · 2.1 · 8.2 · 5.3 · 31.6 · 3.1 · 1.5 · 6.5 · 3.4 · 2.2 · 1.3
Prumnopitys andina [205]	337	1.6 · 2.2 · 1.1 · 18.6 · 1.8 · 0.4 · 0.7 · 0.4 · 1.6 · 5.6 · 12.3 · tr. · 37.1 · 5.1 · 4.1 · 3.5 · 2.3
Prumnopitys taxifolia [205]	338	0.4 · 11.1 · 14.6 · 1.6 · 0.2 · 0.6 · 5.9 · 1.2 · 2.9 · 13.0 · 35.3 · 1.2 · 0.5 · 0.6 · 1.1 · 2.9 · 3.4 · 0.7
Pherosphaera fitzgeraldii [205]	339	0.2 · 1.1 · 7.3 · 17.2 · 12.6 · 0.8 · 1.3 · 1.3 · 1.7 · tr. · 12.2 · 21.9 · 2.5 · 0.9 · 1.7 · 0.9 · 2.8 · 3.0 · 2.3 · 5.3
Dacrydium cupressinum [205]	340	1.8 · 1.8 · 0.6 · 21.0 · 1.8 · 0.4 · 0.8 · 1.4 · 4.6 · 11.4 · 0.5 · 36.1 · 1.1 · 0.3 · 0.3 · 0.4 · 4.1 · 5.2 · 6.7
Agathis robusta [205]	341	2.5 · 1.2 · 1.1 · 19.8 · 1.6 · 0.3 · 1.5 · 0.3 · 1.1 · 9.7 · 5.6 · 41.8 · 0.9 · 0.3 · 0.2 · 0.9 · 0.7 · 0.6
Agathis moorei [205]	342	0.4 · 0.6 · 1.0 · 22.5 · 1.0 · 0.7 · 1.3 · 2.0 · 7.9 · 7.1 · 40.6 · 3.1 · 0.7 · 2.0 · 2.5 · 1.6
Agathis australis [205]	343	3.1 · 0.9 · 1.0 · 13.5 · 1.3 · 0.9 · 1.3 · 6.4 · 8.0 · 27.5 · 3.5 · 3.2 · 1.4 · 12.9 · 4.6 · 6.1 · 4.5
Araucaria montana [205]	344	0.2 · 0.3 · 0.5 · 7.8 · 5.3 · 0.2 · 0.3 · 35.2 · 0.6 · 1.2 · 2.9 · 11.3 · 8.9 · 1.1 · 15.8 · 1.8 · 2.0 · 1.5 · 0.5 · 1.8
Araucaria luxurians [205]	345	0.3 · 5.8 · 21.9 · 6.9 · 0.5 · 6.4 · 1.6 · 4.0 · 10.4 · 30.2 · 0.7 · 2.8 · 1.1 · 0.6 · 3.0 · 0.8 · 3.3
Araucaria cunninghamii [205]	346	0.3 · 0.5 · 25.4 · 4.3 · 2.9 · 2.4 · 0.6 · 4.1 · 1.5 · 4.0 · 4.8 · 12.3 · 2.5 · 4.9 · 4.9 · 5.6 · 3.3 · 1.3 · 4.5
Araucaria angustifolia [205]	347	tr. · 0.9 · 0.8 · 14.7 · 0.3 · 2.3 · 2.4 · 0.4 · 0.7 · 2.3 · 0.7 · 6.4 · 41.0 · 4.3 · 3.1 · 10.1 · 4.1 · 3.3 · 2.0
Araucaria araucana [205]	348	1.2 · 0.4 · 1.2 · 15.5 · 18.8 · 0.8 · 1.3 · 0.5 · 1.0 · 4.5 · 8.9 · 30.1 · 0.9 · 2.6 · 2.6 · 1.4 · 3.9 · 2.7 · 1.4
Keteleeria evelyniana [205]	349	1.1 · 0.6 · 1.6 · 17.3 · 2.0 · 1.0 · 0.5 · 1.9 · 9.1 · 0.2 · 5.5 · 30.9 · 0.4 · 1.9 · 3.0 · 3.1 · 20.0
Abies vejarii [205]	350	5.9 · 3.1 · 1.2 · 12.4 · 1.1 · 0.3 · 0.7 · 3.5 · 1.9 · 7.2 · 1.9 · 9.2 · 2.9 · 18.2 · 11 · 2.0 · 1.0 · 1.1 · 0.7 · 3.9 · 1.5 · 9.0
Abies nordmanniana [208]	351	0.9 · 0.1 · 1.6 · 1.4 · 10.1 · tr. · 0.4 · 1.1 · tr. · 1.3 · 4.5 · 0.5 · 6.6 · 1.1 · 11.9 · 2.2 · 44.4 · 3.3 · 0.3 · 0.2 · 0.6 · 3.4 · 1.0 · 1.4 · 1.7
Abies procera [208]	352	0.1 · tr. · 0.9 · 1.2 · 12.9 · tr. · 0.3 · 0.7 · tr. · 0.1 · 4.1 · 2.9 · 11.3 · 0.8 · 16.7 · 3.3 · 34.4 · 4.1 · 1.0 · 0.1 · 0.4 · 2.0 · 0.5 · 1.1 · 1.1
Abies amabilis [208]	353	0.6 · tr. · 1.6 · 1.0 · 9.7 · tr. · 0.7 · 0.9 · tr. · 0.2 · 4.6 · 1.1 · 6.0 · 1.8 · 12.7 · 3.7 · 42.5 · 4.4 · 0.2 · 0.2 · 0.4 · 3.4 · 0.5 · 2.0 · 1.8
Abies concolor [205]	354	2.3 · 0.7 · 1.0 · 11.1 · 1.3 · 0.2 · 0.5 · 3.4 · 1.0 · 9.9 · 1.1 · 7.9 · 2.2 · 32.2 · 3.4 · 0.9 · 0.4 · 0.4 · 0.4 · 4.1 · 2.0 · 13.4
Abies sachalinensis [205]	355	9.6 · 5.6 · 2.6 · 12.4 · 1.7 · 0.5 · 1.0 · 4.9 · 1.9 · 10.4 · 4.2 · 10.0 · 4.6 · 10.3 · 3.6 · 1.0 · 0.5 · 0.4 · 0.2 · 2.7 · 1.2 · 10.9
Abies veitchii [205]	356	1.3 · 1.0 · 1.4 · 13.6 · 4.7 · 0.7 · 0.8 · 5.5 · 4.6 · 3.2 · 3.2 · 14.0 · 4.7 · 11.6 · 3.6 · 2.4 · 2.0 · 1.0 · 3.9 · 1.2 · 8.7
Abies cephalonica [205]	357	5.5 · 2.0 · 1.3 · 1.9 · 0.5 · 0.5 · 0.8 · 3.1 · 1.8 · 6.0 · 1.7 · 5.8 · 1.5 · 28.1 · 3.1 · 0.4 · tr. · 0.9 · 2.4 · 2.7 · 22.1
Abies pinsapo [205]	358	0.4 · 0.8 · 1.1 · 13.0 · 0.7 · 2.0 · 2.2 · 11.7 · 2.1 · 6.0 · 1.1 · 25.5 · 1.8 · 2.2 · 1.1 · 1.1 · 0.3 · 3.2 · 1.8 · 21.8
Abies grandis [205]	359	1.0 · 0.8 · 1.2 · 3.5 · 1.0 · 0.2 · 0.8 · 5.3 · 1.5 · 8.9 · 3 · 5.7 · 2.6 · 24.6 · 4.9 · 1.6 · 0.7 · 1.9 · 0.9 · 3.9 · 2.5 · 12.9
Abies alba [205]	360	1.2 · 1.0 · 1.9 · 12.0 · 0.9 · 0.7 · 1.3 · 3.6 · 2.7 · 10.8 · 1.7 · 8.6 · 2.0 · 22.6 · 3.3 · 1.6 · 2.1 · 3.9 · 1.5 · 16.6
Tsuga canadensis [208]	361	0.3 · tr. · 1.5 · 0.4 · 12.2 · tr. · 0.2 · 0.7 · tr. · 0.3 · 1.5 · 2.2 · 2.9 · 0.1 · 11.4 · 1.1 · 51.2 · 0.7 · 1.0 · 0.2 · 0.4 · 6.1 · 0.3 · 3.2 · 2.1
Tsuga heterophylla [208]	362	0.2 · tr. · 1.7 · 0.6 · 15.9 · tr. · 0.5 · 1.0 · tr. · 0.2 · 2.6 · 1.6 · 4.4 · 0.3 · 9.0 · 1.8 · 49.7 · 2.4 · 1.1 · 0.4 · 0.3 · 2.7 · 0.6 · 1.9 · 1.1
Pseudotsuga sinensis var. wilsoniana [208]	363	0.1 · 26.5 · 2.0 · 0.2 · 2.5 · 52.9 · 16.4 · 0.9 · 0.3 · 0.2
Pseudotsuga macrocarpa [205]	364	1.8 · 2.4 · 0.9 · 12.2 · 1.2 · 0.2 · 1.9 · 1.3 · 0.8 · 4.1 · 0.6 · 7.1 · 1.0 · 36.7 · 3.6 · 3.2 · 0.8 · 0.3 · 4.2 · 3.7 · 11.9
Pseudotsuga menziesii [205]	365	3.8 · 2.4 · 1.1 · 10.8 · 1.3 · 0.3 · 1.3 · 2.1 · 0.9 · 2.8 · 0.4 · 10.5 · 1.4 · 16.5 · 6.1 · 5.4 · 0.6 · 1.6 · 6.2 · 1.6 · 22.6
Pseudolarix amabilis [205]	366	1.7 · 1.7 · 0.6 · 18.5 · 2.4 · 0.9 · 1.7 · 0.9 · 1.3 · 3.4 · 2 · 8.7 · 4.2 · 31.7 · 4.7 · 0.3 · 6.2 · 6.3
Pinus strobiformis [205]	367	4.8 · 4.8 · 0.9 · 11.5 · 1.6 · 0.3 · 1.6 · 0.7 · 1.1 · 4.0 · 0.4 · 9.5 · 1.3 · 27.5 · 1.4 · 3.9 · 0.3 · tr. · 0.9 · 3.9 · 3.9 · 14.6
Pinus monophylla [205]	368	4.3 · 1.9 · 0.5 · 11.9 · 1.2 · 0.2 · 2.0 · tr. · 1.3 · 10.2 · 0.9 · 15.1 · 1.9 · 27.2 · 0.9 · 3.6 · 0.2 · tr. · 1.0 · 4.1 · 2.1 · 7.5
Pinus koraiensis [210]	369	12.2 · 1.7 · 0.2 · 14.0 · 0.7 · 0.7 · 2.5 · 2.3 · 4.4 · 0.8 · 0.8 · 16.3 · 3.4 · 25.1 · 1.7 · 1.1 · 0.5 · 5.7 · 1 · 3.2
Pinus pinaster [205]	370	8.3 · 1.9 · 0.8 · 8.7 · 0.8 · 0.8 · 0.5 · 0.4 · 2.1 · 5.3 · 0.2 · 12.6 · 16.0 · 23.4 · 1.1 · 6.2 · 1.6 · 8.5
Pinus halepensis [205]	371	5.1 · 1.4 · 0.9 · 13.3 · 2.0 · 0.2 · 4.2 · 0.4 · 2.1 · 2.6 · 9.8 · 33.2 · 2.4 · 6.4 · 0.8 · 0.6 · 0.9 · 5.9 · 1.1 · 5.9
Pinus bungeana [205]	372	6.0 · 1.5 · 0.2 · 11.5 · 1.4 · 0.2 · 2.0 · 0.3 · 0.9 · 10.8 · 0.8 · 16.4 · 2.3 · 25.6 · 1.7 · 1.2 · 1.1 · 0.5 · 1.6 · 4.8 · 0.9 · 5.4 · 0.6
Pinus aristata [205]	373	2.5 · 1.4 · 1.2 · 12.7 · 1.7 · 0.3 · 2.1 · 0.4 · 1.2 · 4.0 · 0.5 · 13.4 · 2.2 · 29.0 · 1.9 · 8.4 · 0.2 · tr. · 1.1 · 4.8 · 1.7 · 9.1
Pinus nigra [208]	374	8.7 · 0.2 · 7.9 · 0.2 · 11.2 · tr. · 0.7 · 0.8 · 0.2 · 4.0 · 0.7 · 0.9 · 2.7 · 0.4 · 8.2 · 1.9 · 35.0 · 1.9 · 0.5 · 0.4 · 0.7 · 6.7 · 0.6 · 1.3 · 4.3
Pinus cembra [205]	375	2.5 · 2.5 · 2.2 · 13.3 · 0.9 · 1.1 · 1.4 · 2.2 · 1.2 · 3.6 · 0.8 · 8.4 · 2.2 · 29.1 · 3.2 · 5.0 · 0.7 · 0.7 · 1.2 · 5.7 · 3.2 · 8.9

Species	No.	Data
Pinus ponderosa [207]	376	[illegible]
Pinus jeffreyi [205]	377	[illegible]
Pinus resinosa [205]	378	[illegible]
Pinus palustris [205]	379	[illegible]
Pinus sylvestris [208]	380	[illegible]
Pinus pinea [205]	381	[illegible]
Pinus wallichiana [205]	382	[illegible]
Pinus contorta [208]	383	[illegible]
Picea obovata [208]	384	[illegible]
Picea orientalis [208]	385	[illegible]
Picea asperata [205]	386	[illegible]
Picea chihuahuana [205]	387	[illegible]
Picea torano [205]	388	[illegible]
Picea omorica [205]	389	[illegible]
Picea mariana [208]	390	[illegible]
Picea engelmannii [208]	391	[illegible]
Picea sitchensis [208]	392	[illegible]
Picea pungens [210]	393	[illegible]
Picea glauca [205]	394	[illegible]
Picea abies [208]	395	[illegible]
Larix gmelinii [211]	396	[illegible]
Larix lyallii [205]	397	[illegible]
Larix decidua [208]	398	[illegible]
Larix sibirica [210]	399	[illegible]
Larix kaempferi [211]	400	[illegible]
Larix laricina [205]	401	[illegible]
Cedrus atlantica [205]	402	[illegible]
Cedrus libani [205]	403	[illegible]
Cedrus deodara [208]	404	[illegible]
Sciadopitys verticillata [205]	405	[illegible]
Torreya californica [205]	406	[illegible]
Cephalotaxus sinensis [205]	407	[illegible]
Cephalotaxus fortunei [205]	408	[illegible]
Cephalotaxus harringtonii [205]	409	[illegible]
Taxus cuspidata [205]	410	[illegible]
Taxus brevifolia [205]	411	[illegible]
Taxus baccata [205]	412	[illegible]
Callitris preissii [205]	413	[illegible]
Widdringtonia schwarzii [205]	414	[illegible]
Hesperocyparis bakeri [205]	415	[illegible]
Hesperocyparis lusitanica [205]	416	[illegible]
Hesperocyparis goveniana [205]	417	[illegible]
Austrocedrus chilensis [205]	418	[illegible]
Sequoiadendron giganteum [205]	419	[illegible]
Callitropsis nootkatensis [208]	420	[illegible]
Callitropsis funebris [205]	421	[illegible]
Platycladus orientalis [205]	422	[illegible]
Chamaecyparis thyoides [208]	423	[illegible]
Chamaecyparis pisifera [208]	424	[illegible]
Chamaecyparis hodginsii [205]	425	[illegible]
Chamaecyparis lawsoniana [208]	426	[illegible]
Taiwania cryptomerioides [205]	427	[illegible]
Juniperus sabina [205]	428	[illegible]
Juniperus communis [208]	429	[illegible]
Juniperus chinensis [205]	430	[illegible]
Juniperus virginiana [208]	431	[illegible]
Taxodium mucronatum [205]	432	[illegible]
Taxodium distichum var. distichum [205]	433	[illegible]
Taxodium distichum var. imbricatum [205]	434	[illegible]
Sequoia sempervirens [205]	435	[illegible]
Cunninghamia lanceolata [205]	436	[illegible]
Cunninghamia lanceolata var. konishii [205]	437	[illegible]
Athrotaxis laxifolia [205]	438	[illegible]
Thujopsis dolabrata [205]	439	[illegible]
Tetraclinis articulata [205]	440	[illegible]
Microbiota decussata [205]	441	[illegible]
Diselma archeri [205]	442	[illegible]
Cupressus torulosa [205]	443	[illegible]
Cupressus dupreziana [205]	444	[illegible]
Cupressus sempervirens [208]	445	[illegible]
Calocedrus decurrens [205]	446	[illegible]
Metasequoia glyptostroboides [205]	447	[illegible]
Cryptomeria japonica [205]	448	[illegible]
Thuja koraiensis [205]	449	[illegible]
Thuja standishii [205]	450	[illegible]
Thuja occidentalis [205]	451	[illegible]
Thuja plicata [208]	452	[illegible]
Cyathea dealbata [212]	453	[illegible]
Hymenophyllum plicatum [213]	454	[illegible]
Hymenophyllum caudiculatum [213]	455	[illegible]
Ptisana salicina [214]	456	[illegible]
Salvinia natans [214]	457	[illegible]
Azolla caroliniana [215]	458	[illegible]
Lygodium japonicum [216]	459	[illegible]
Claytosmunda claytoniana [212]	460	[illegible]
Osmunda regalis [217]	461	[illegible]
Osmundastrum cinnamomeum [216]	462	[illegible]
Woodsia glabella [218]	463	[illegible]
Amauropelta noveboracensis [212]	464	[illegible]
Phegopteris connectilis [212]	465	[illegible]
Pyrrosia eleagnifolia [212]	466	[illegible]
Platycerium bifurcatum [102]	467	[illegible]
Pleopeltis polypodioides [216]	468	[illegible]
Polypodium vulgare [219]	469	[illegible]
Gymnocarpium dryopteris [218]	470	[illegible]
Cystopteris fragilis [212]	471	[illegible]
Deparia pycnosora [212]	472	[illegible]
[]*Athyrium sinense* [212]	473	[illegible]
Athyrium distentifolium [218]	474	[illegible]
Athyrium spinulosum [212]	475	[illegible]
Athyrium crenulatoserrulatum [212]	476	[illegible]
Athyrium yokoscense [212]	477	[illegible]
Athyrium filix0femina [212]	478	[illegible]
Pteridium esculentum [212]	479	[illegible]
Pteridium aquilinum [220]	480	[illegible]
Asplenium oblongifolium [212]	481	[illegible]
Asplenium trichomanes [102]	482	[illegible]
Asplenium scolopendrium [41]	483	[illegible]
Asplenium nidus [102]	484	[illegible]
Adiantum pedatum [212]	485	[illegible]
Polystichum tripteron [212]	486	[illegible]
Dryopteris austriaca [221]	487	[illegible]
Dryopteris expansa [218]	488	[illegible]
Dryopteris goeringiana [212]	489	[illegible]
Dryopteris crassirhizoma [212]	490	[illegible]
Dryopteris filix-mas [221]	491	[illegible]
Onoclea sensibilis [212]	492	[illegible]
Matteuccia struthiopteris [222]	493	[illegible]
Equisetum fluviatile [217]	494	[illegible]
Equisetum variegatum [223]	495	[illegible]
Equisetum hyemale [224]	496	[illegible]
Equisetum scirpoides [223]	497	[illegible]
Equisetum arvense [223]	498	[illegible]
Psilotum nudum [217]	499	[illegible]
Riccia fluitans [96]	500	[illegible]
Conocephalum conicum [26]	501	[illegible]
Marchantia polymorpha [26]	502	[illegible]
Metzgeria pubescens [26]	503	[illegible]
Ptilidium ciliare [26]	504	[illegible]
Plagiochila porelloides [26]	505	[illegible]
Pellia neesiana [96]	506	[illegible]
Sphagnum majus [225]	507	[illegible]
Sphagnum nemoreum [226]	508	[illegible]
Sphagnum squarrosum [226]	509	[illegible]
Sphagnum magellanicum [225]	510	[illegible]
Sphagnum fimbriatum [225]	511	[illegible]
Aulacomnium palustre [226]	512	[illegible]
Tortella tortuosa [227]	513	[illegible]
Philonotis fontana [226]	514	[illegible]
Entodon schleicheri [26]	515	[illegible]
Thuidium assimile [26]	516	[illegible]
Calliergon cordifolium [96]	517	[illegible]
Hygrohypnella ochracea [226]	518	[illegible]
Helodium blandowii [226]	519	[illegible]
Rhytidium rugosum [26]	520	[illegible]
Drepanocladus lycopodioides [96]	521	[illegible]
Amblystegium riparium [228]	522	[illegible]
Cratoneuron filicinum [26]	523	[illegible]
Pseudanomodon attenuatus [26]	524	[illegible]
Neckera pennata [26]	525	[illegible]
Ctenidium molluscum [227]	526	[illegible]
Climacium dendroides [226]	527	[illegible]
Fontinalis antipyretica [229]	528	[illegible]
Hypnum andoi [230]	529	[illegible]
Hypnum jutlandicum [231]	530	[illegible]
Hypnum cupressiforme [232]	531	[illegible]
Rhytidiadelphus squarrosus [233]	532	[illegible]
Kindbergia praelonga [234]	533	[illegible]
Eurhynchium praelongum [228]	534	[illegible]
Eurhynchium striatum [233]	535	[illegible]
Rhynchostegium murale [228]	536	[illegible]
Brachythecium erythrorrhizon [228]	537	[illegible]
Brachythecium rivulare [26]	538	[illegible]
Brachythecium rutabulum [231]	539	[illegible]
Calliergonella cuspidata [235]	540	[illegible]
Bryum sp. [226]	541	[illegible]
Ptychostomum moravicum [231]	542	[illegible]
Ptychostomum pseudotriquetrum [226]	543	[illegible]
Rhodobryum ontariense [236]	544	[illegible]
Pseudobryum cinclidioides [226]	545	[illegible]
Plagiomnium confertidens [26]	546	[illegible]
Plagiomnium medium [226]	547	[illegible]
Plagiomnium cuspidatum [237]	548	[illegible]
Mnium hornum [238]	549	[illegible]
Ceratodon purpureus [239]	550	[illegible]
Dichodontium pellucidum [227]	551	[illegible]
Funaria hygrometrica [240]	552	[illegible]
Physcomitrium patens [24]	553	[illegible]
Pogonatum urnigerum [227]	554	[illegible]
Atrichum undulatum [230]	555	[illegible]
Polytrichum juniperinum [226]	556	[illegible]
Polytrichum commune [26]	557	[illegible]

* – the first publisher data
