# Supplementary material for: Phylogenetic Aspects of Higher Plant Lipid Fatty Acid Profile
Source: Int J Mol Sci. 2025 Sep 26;26(19):9424. doi: 10.3390/ijms26199424 (PMC12524838; doi:10.3390/ijms26199424)
Supplement: Supplementary file 1 [file ijms-26-09424-s001.zip › Table_S2.pdf]

Table S2. Integral characteristics of lipid fatty acids (FAs) composition of Embryophyta (number, acyl chain length (ACL), double bond content, and unsaturation index (UI))

Species	№	Number of iFA	FA content with a certain acyl chain length (%)				ACL	Acyl chain length (%)		FA by number of double bonds in the acyl chain (%)							UI
			shot	medium	long	very-long		even	odd	saturated	monoene	diene	triene	tetraene	pentaene	hexaene	
Carissa edulis [119]	1	6	-	-	99.9	0.1	17.6	100.0	-	33.0	28.3	38.7	-	-	-	-	1.1
Lomelosia cretica [112]	2	16	-	-	81.8	18.2	18.1	98.2	1.8	49.2	4.8	10.2	33.9	1.9	-	-	1.3
Aristolochia fontanesii [121]	3	10	-	6.1	91.4	2.5	17.1	100.0	-	43.0	13.1	18.6	25.3	-	-	-	1.3
Strelitzia reginae [122]	4	10	-	0.1	94.4	1.0	17.4	95.5	-	32.5	5.1	17.1	40.8	-	-	-	1.6
Cistus ladanifer [123]	5	14	-	0.2	58.9	40.9	18.7	93.4	6.6	66.8	10.1	13.3	9.8	-	-	-	0.7
Abelmoschus esculentus [119]	6	8	-	-	99.7	0.3	17.2	100.0	-	46.3	22.6	30.9	0.2	-	-	-	0.9
Malvella sherardiana [124]	7	9	-	0.9	92.7	4.7	17.4	98.3	-	64.0	15.5	15.6	3.2	-	-	-	0.6
Hibiscus cannabinus [125]	8	12	-	-	98.7	1.5	17.6	100.0	0.2	25.1	3.3	13.2	58.6	-	-	-	2.1
Hibiscus sabdariffa [124]	9	10	-	-	98.5	1.5	17.6	100.0	-	23.3	3.8	14.9	58.0	-	-	-	2.1
Malva neglecta [124]	10	10	-	1.6	89.3	4.1	16.9	93.4	1.6	55.5	8.5	17.8	13.2	-	-	-	0.8
Malva sylvestris [126]	11	21	tr.	0.1	95.1	4.8	17.9	97.2	2.8	16.5	3.7	12.1	67.7	-	-	-	2.3
Glyphaea brevis [127]	12	23	-	-	99.7	0.3	17.5	100.0	-	29.0	1.6	17.2	52.2	tr.	tr.	tr.	1.9
Corchorus olitorius [125]	13	13	-	-	98.2	1.9	17.6	100.1	-	26.1	4.6	13.6	55.8	-	-	-	1.9
Adansonia digitata [119]	14	8	-	-	99.8	0.2	17.0	100.0	-	52.6	13.1	18.9	15.4	-	-	-	1.0
Sterculia tragacantha [128]	15	27	-	0.8	94.6	4.3	17.2	95.7	4.0	40.8	14.2	9.7	34.4	0.2	0.3	0.1	1.4
Dioscorea communis [129]	16	26	-	0.2	96.0	4.1	17.9	99.7	0.6	20.3	5.1	42.7	32.2	-	tr.	-	1.9
Raphia sudanica [130]	17	13	-	7.4	93.1	tr.	16.8	100.1	0.4	42.4	14.8	41.3	2.0	-	-	-	1.0
Raphia regalis [130]	18	13	-	0.8	96.3	tr.	17.3	96.4	0.7	36.5	11.3	32.2	17.1	-	-	-	1.3
Raphia vinifera [130]	19	13	-	4.2	95.9	tr.	16.9	99.4	0.7	41.7	19.6	35.5	3.3	-	-	-	1.0
Raphia hookeri [130]	20	13	-	8.6	91.3	tr.	16.3	99.9	tr.	47.8	3.5	42.0	6.6	-	-	-	1.1
Elaeis guineensis [131]	21	10	-	0.2	92.0	20.3	17.9	112.2	0.3	49.0	7.2	35.4	20.9	-	-	-	1.4
Papaver rhoeas [120]	22	17	-	-	92.9	7.2	17.6	92.7	7.4	34.8	8.2	14.6	42.5	-	-	-	1.7
Vitis vinifera [132]	23	23	-	1.8	94.7	3.8	17.4	97.8	2.5	37.0	3.4	7.5	52.2	0.2	-	-	1.8
Arum elongatum [133]	24	19	0.1	0.5	94.7	2.7	17.7	97.8	0.2	22.3	19.9	34.4	21.4	-	-	-	1.5
Xanthosoma sagittifolium [125]	25	14	-	-	93.2	7.7	17.7	100.9	-	37.5	6.1	22.8	34.5	-	-	-	1.6
Pleroma granulosum [134]	26	7	-	-	77.9	18.6	17.9	96.5	-	45.3	41.3	9.9	-	-	-	-	0.6
Lumnitzera racemosa [135]	27	14	-	9.7	88.2	1.0	15.8	95.5	2.7	82.5	10.5	2.7	2.6	-	-	-	0.2
Eucalyptus accedens [136]	28	14	-	-	88.8	10.2	17.9	98.7	0.3	40.1	17.7	37.1	4.1	-	-	-	1.0
Eucalyptus patens [136]	29	15	-	-	94.8	3.8	17.7	98.4	0.2	29.1	18.7	44.3	5.9	0.6	-	-	1.3
Eucalyptus wandoo [136]	30	18	-	1.1	91.4	15.2	17.9	107.7	-	42.5	13.4	45.1	4.6	2.1	-	-	1.3
Eucalyptus marginata [136]	31	16	-	-	94.4	4.7	17.6	98.6	0.5	39.0	15.3	40.7	4.0	0.1	-	-	1.1
Eucalyptus diversicolor [136]	32	11	-	-	93.2	6.7	17.8	99.8	-	27.2	18.2	48.0	6.4	-	-	-	1.3
Myrtus communis [137]	33	7	-	4.3	93.8	-	17.3	97.6	0.5	25.7	72.4	-	-	-	-	-	0.7
Corymbia calophylla [136]	34	16	-	1.5	91.3	5.1	17.6	97.6	0.3	31.4	15.5	38.2	12.7	0.1	-	-	1.3
Punica granatum [132]	35	21	-	3.1	95.1	1.6	17.2	93.9	5.9	34.6	14.7	17.9	32.6	-	-	-	1.5
Juglans regia [132]	36	24	-	2.0	87.1	10.9	17.7	97.8	2.2	56.6	6.4	5.4	27.8	3.8	-	-	1.2
Quercus ilex [138]	37	11	-	-	97.9	2.6	17.9	99.7	0.8	14.4	66.2	16.5	3.4	-	-	-	1.1
Castanea sativa [139]	38	21	0.2	1.2	69.4	19.3	18.2	88.7	1.4	41.5	6.4	20.9	21.3	-	-	-	1.1
Cynoglossum creticum [120]	39	16	-	-	78.9	21.1	18.4	98.6	1.4	38.6	13.9	9.6	36.1	1.8	-	-	1.5
Anchusa azurea [129]	40	24	-	0.2	93.7	6.2	17.9	99.5	0.6	16.8	3.2	12.2	67.9	-	-	-	2.3
Anchusa officinalis [133]	41	18	0.1	0.8	94.5	4.6	17.3	99.5	0.5	50.8	36.1	10.0	3.1	-	-	-	0.7
Borago officinalis [140]	42	8	-	-	100.0	-	17.6	100.0	-	19.7	6.6	12.7	45.8	15.2	-	-	2.3
Lilium longiflorum *	43	21	-	0.2	97.0	2.8	17.7	98.1	1.9	27.1	7.7	12.1	53.1	-	-	-	1.9
Asparagus acutifolius [129]	44	15	-	1.1	94.6	4.4	17.5	98.3	1.8	38.8	4.9	42.3	14.1	-	-	-	1.3
Xanthorrhoea preissii [122]	45	9	-	0.6	57.6	9.3	18.2	67.5	-	21.0	20.5	12.5	13.5	-	-	-	0.9
Eremurus spectabilis [133]	46	18	-	1.3	89.0	9.4	17.4	98.6	1.1	55.9	19.1	9.6	15.1	-	-	-	0.8
Muscari comosum [120]	47	10	-	-	91.3	8.7	17.9	99.3	0.7	30.2	10.8	47.7	11.3	-	-	-	1.4
Allium orientale [133]	48	19	0.2	1.1	92.1	6.7	17.4	99.4	0.7	52.1	36.8	8.1	3.1	-	-	-	0.6
Allium schoenoprasum [120]	49	15	-	-	96.2	3.8	17.9	99.4	0.6	20.3	5.7	17.9	56.1	-	-	-	2.1
Allium sativum [133]	50	12	-	-	83.7	1.5	17.6	84.4	0.8	21.9	4.8	53.6	4.5	-	0.4	-	1.3
Allium ampeloprasum [141]	51	12	-	0.2	66.4	1.7	18.1	67.9	0.4	2.9	5.8	52.0	7.6	-	-	-	1.3
Allium cepa [141]	52	12	-	0.6	84.9	2.8	17.6	86.6	1.7	24.5	14.3	46.9	2.6	-	-	-	1.2
Carex depressa [142]	53	9	-	-	91.1	7.1	16.9	98.2	-	75.5	4.9	11.6	6.2	-	-	-	0.5
Carex cilicica [142]	54	9	-	-	92.1	5.0	16.9	97.1	-	65.7	2.1	23.4	5.9	-	-	-	0.7
Carex halleriana [142]	55	9	-	-	85.9	9.1	17.1	95.0	-	72.6	6.7	7.2	8.5	-	-	-	0.5
Carex hartmanii [142]	56	9	-	-	99.4	2.6	16.4	102.0	-	86.7	5.2	6.7	3.4	-	-	-	0.3
Carex orbicularis [142]	57	9	-	-	96.7	3.2	16.6	99.9	-	78.8	3.2	8.7	9.2	-	-	-	0.5
Carex punctata [142]	58	9	-	-	94.3	5.7	16.6	100.0	-	91.6	3.5	3.1	1.8	-	-	-	0.2
Carex pendula [142]	59	9	-	-	97.5	2.5	16.8	100.0	-	69.4	3.7	12.8	14.1	-	-	-	0.7
Carex sylvatica [142]	60	9	-	-	96.3	3.7	16.6	100.0	-	84.5	4.5	6.3	4.7	-	-	-	0.3
Carex rostrata [142]	61	9	-	-	96.5	3.6	16.5	100.1	-	87.9	4.2	3.9	4.1	-	-	-	0.2
Carex liparocarpus [142]	62	9	-	-	95.9	5.1	17.1	101.0	-	64.1	6.1	15.4	15.4	-	-	-	0.8

Carex paniculata [142]	63	9	-	-	94.8	5.2	16.7	100.0	-	83.7	2.8	7.9	5.6	-	-	-	0.4
Carex pallescens [142]	64	9	-	-	94.1	6.2	16.9	100.3	-	75.9	4.4	11.7	8.3	-	-	-	0.5
Carex flacca [142]	65	9	-	-	96.3	3.9	16.8	100.2	-	74.9	6.1	12.5	6.7	-	-	-	0.5
Carex digitata [142]	66	9	-	-	96.7	5.3	16.8	102.0	-	79.2	6.1	9.5	7.2	-	-	-	0.5
Carex brevicollis [142]	67	9	-	-	96.3	3.7	16.9	100.0	-	66.3	4.2	18.1	11.4	-	-	-	0.8
Zea mays [143]	68	4	-	5.7	88.0	-	15.9	93.7	-	51.8	41.9	-	-	-	-	-	0.4
Leucopogon conostephioides [122]	69	8	-	-	89.6	2.2	17.5	91.8	-	27.0	17.0	25.6	22.2	-	-	-	1.3
Vaccinium myrtillus [144]	70	12	-	-	85.1	14.0	18.1	96.0	3.1	38.8	12.5	30.6	17.2	-	-	-	1.3
Arbutus unedo [145]	71	8	-	-	93.3	1.3	17.3	94.6	-	34.9	7.6	7.9	44.2	-	-	-	1.6
Arbutus andrachne [145]	72	8	-	-	93.8	1.5	17.4	95.3	-	29.4	10.5	13.0	42.4	-	-	-	1.6
Labisia pumila var. pumila [146]	73	6	-	-	100.0	-	17.4	100.0	-	31.6	21.1	28.7	18.5	-	-	-	1.3
Labisia pumila var. alata [146]	74	6	-	-	100.0	-	17.4	100.0	-	32.9	21.1	24.9	21.1	-	-	-	1.3
Labisia pumila var. lanceolata [146]	75	6	-	-	100.0	-	17.5	100.0	-	28.3	18.7	28.9	24.1	-	-	-	1.5
Aegiceras corniculatum [135]	76	10	-	37.8	55.8	1.4	14.8	93.7	1.3	76.7	0.7	12.1	5.5	-	-	-	0.4
Camellia Sinensis [147]	77	12	-	5.6	91.3	0.6	16.9	97.5	-	46.7	25.4	12.1	13.3	-	-	-	0.9
Eucommia ulmoides [148]	78	30	-	0.1	49.1	50.9	19.3	99.4	0.7	38.9	33.6	7.8	2.1	11.1	6.6	tr.	1.3
Artemisia lercheana [16]	79	15	-	-	72.1	28.0	18.8	94.1	6.0	41.8	7.1	21.6	29.6	-	-	-	1.4
Tragopogon sinuatus [120]	80	18	-	-	95.0	5.0	17.7	96.4	3.6	28.6	6.0	15.1	50.3	-	-	-	1.9
Crepis vesicaria [120]	81	17	-	-	95.5	4.5	17.6	95.9	4.1	30.3	6.1	18.3	43.7	1.6	-	-	1.8
Helichrysum stoechas [139]	82	22	0.2	12.4	80.0	7.4	16.8	97.8	2.2	44.3	7.0	25.9	22.8	-	-	-	1.3
Arctotheca calendula [122]	83	9	-	3.1	87.3	tr.	17.5	90.4	-	20.0	16.3	50.4	3.7	-	-	-	1.3
Symphyotrichum novi-belgii [122]	84	8	-	0.7	94.6	tr.	17.2	95.3	-	35.5	16.1	13.5	30.2	-	-	-	1.3
Helminthotheca echioides [122]	85	8	-	8.4	65.8	1.6	17.0	75.8	-	23.5	8.1	28.3	15.9	-	-	-	1.1
Gundelia tournefortii [149]	86	7	-	-	98.0	0.3	17.8	98.3	-	10.9	29.5	57.8	0.1	-	-	-	1.5
Stizolophus balsamita [150]	87	20	-	0.9	99.2	tr.	17.4	98.8	1.3	33.7	11.4	39.6	15.4	-	-	-	1.4
Matricaria chamomilla var. recutita [139]	88	22	0.2	0.8	94.5	4.5	17.8	98.1	1.9	23.8	8.4	44.9	22.9	-	-	-	1.7
Urospermum dalechampii [122]	89	8	-	6.4	85.7	tr.	16.9	92.1	-	32.3	8.7	17.2	33.9	-	-	-	1.4
Scolymus hispanicus [120]	90	16	-	-	96.3	5.9	17.6	99.5	2.7	33.8	13.7	28.1	26.6	-	-	-	1.5
Dahlia variabilis [120]	91	20	0.9	2.6	89.2	7.3	17.3	98.2	1.8	46.7	7.2	36.9	9.2	-	-	-	1.1
Silybum marianum [129]	92	12	-	0.7	93.5	5.8	17.6	97.7	2.3	43.5	3.9	31.0	21.6	-	-	-	1.3
Gymnanthemum amygdalinum [151]	93	12	-	0.9	65.6	7.6	17.6	73.5	0.6	34.1	2.3	15.8	21.9	-	-	-	1.0
Rhanterium adpressum [152]	94	12	-	1.9	84.6	13.4	17.3	97.7	2.2	80.4	12.9	5.5	1.1	-	-	-	0.3
Chondrilla juncea [129]	95	21	tr.	0.2	94.7	5.1	17.9	98.9	1.1	21.5	2.1	19.9	56.5	-	-	-	2.1
Hypochaeris cretensis [120]	96	18	-	-	95.3	6.0	17.6	96.4	4.9	29.5	8.5	16.4	46.9	-	-	-	1.8
Telekia speciosa [153]	97	4	-	-	83.4	6.5	17.7	89.9	-	26.9	25.9	37.1	-	-	-	-	1.0
Dittrichia viscosa [154]	98	8	-	-	90.8	9.2	18.0	100.0	-	34.6	6.6	19.6	39.2	-	-	-	1.6
Stevia rebaudiana [155]	99	6	-	-	68.3	-	17.2	68.3	-	28.7	5.6	12.4	21.6	-	-	-	0.9
Taraxacum obovatum [129]	100	22	-	0.6	95.9	3.6	17.8	98.9	1.2	19.9	3.9	17.6	58.7	-	-	-	2.2
Taraxacum officinale [144]	101	13	-	0.6	90.1	9.4	17.7	96.8	3.2	48.3	6.3	20.8	24.6	-	-	-	1.2
Sonchus oleraceus [129]	102	15	0.4	0.4	95.5	4.5	17.8	99.7	1.1	19.9	0.9	13.7	66.3	-	-	-	2.3
Reichardia picroides [120]	103	13	-	-	91.3	8.7	17.9	97.3	2.7	34.7	3.1	14.3	47.9	-	-	-	1.8
Centaurea pulcherrima [156]	104	30	-	2.0	94.3	3.7	17.4	96.5	3.5	33.6	15.8	30.0	17.5	1.5	0.7	0.9	1.4
Centaurea pseudoscabiosa [156]	105	30	-	2.0	95.1	2.9	17.3	96.8	3.2	34.1	20.8	30.3	12.9	1.5	0.3	0.1	1.3
Centaurea triumfettii [157]	106	20	-	0.5	99.5	tr.	17.5	99.5	0.5	25.0	31.1	35.1	8.8	-	-	-	1.3
Centaurea tchihatcheffii [158]	107	30	-	3.3	92.9	3.9	17.5	94.2	5.9	27.0	10.3	34.6	25.7	1.6	0.3	0.6	1.7
Centaurea saligna [159]	108	16	0.2	0.9	57.6	9.3	17.7	66.6	1.4	27.3	20.1	11.5	7.2	-	1.9	-	0.7
Centaurea iberica [159]	109	20	-	2.1	97.4	0.6	17.3	98.8	1.3	31.9	9.4	31.2	27.6	-	-	-	1.5
Centaurea calolepis [150]	110	20	-	1.3	98.7	0.1	17.4	99.1	1.0	28.9	20.9	35.9	14.4	-	-	-	1.4
Centaurea babylonica [156]	111	30	-	2.1	93.8	4.1	17.3	96.6	3.4	43.1	12.4	20.3	20.9	2.5	0.6	0.2	1.3
Centaurea solstitialis [157]	112	20	-	1.4	98.5	0.1	17.2	99.1	0.9	37.8	9.0	39.8	13.4	-	-	-	1.3
Centaurea carduiformis [150]	113	20	-	2.2	97.8	tr.	17.3	98.8	1.2	27.8	20.8	40.6	10.8	-	-	-	1.4
Centaurea virgata [157]	114	20	-	3.6	96.4	0.1	17.1	98.5	1.6	39.8	9.7	29.2	21.4	-	-	-	1.3
Centaurea paniculata [139]	115	21	0.1	1.9	90.7	7.3	17.7	99.2	0.8	31.8	18.5	35.0	14.7	-	-	-	1.3
Centaurea cariensis [150]	116	20	-	4.0	95.9	0.1	17.1	98.8	1.2	37.5	16.3	23.9	22.3	-	-	-	1.3
Centaurea aggregata [159]	117	17	0.4	0.6	55.5	10.1	16.9	63.3	3.3	50.9	3.4	0.4	9.2	-	2.7	-	0.4
Centaurea patula [158]	118	30	-	1.7	93.9	4.5	17.5	98.4	1.7	30.9	12.8	19.1	34.2	1.9	0.2	1.0	1.7
Centaurea pterocaula [157]	119	20	-	1.9	98.1	0.1	17.2	98.8	1.3	32.9	17.2	33.3	16.7	-	-	-	1.3
Centaurea depressa [159]	120	19	0.2	0.4	50.4	19.9	17.8	69.7	1.2	28.7	21.8	13.6	5.7	-	1.1	-	0.7
Centaurea pulchella [158]	121	30	-	1.4	93.8	3.9	17.5	97.5	1.6	25.7	2.6	34.2	33.9	1.6	0.3	0.8	1.9
Centaurea kotschyi [157]	122	20	-	0.6	99.3	0.1	17.7	99.4	0.6	16.9	22.6	55.8	5.2	-	-	-	1.5
Centaurea behen [159]	123	19	0.3	1.2	53.4	10.6	17.4	63.2	2.3	38.6	10.5	8.2	6.7	-	1.5	-	0.5
Centaurea cyanus [160]	124	20	0.2	0.2	57.5	42.1	18.6	98.7	1.3	36.2	10.9	6.7	19.3	-	26.9	-	2.2
Calendula officinalis [160]	125	19	0.3	2.2	100.4	2.5	17.0	104.8	0.6	46.1	1.8	20.4	37.1	-	-	-	1.5
Cichorium spinosum [120]	126	17	-	-	94.9	5.1	17.6	94.8	5.2	32.3	6.9	18.6	42.2	-	-	-	1.7
Cichorium intybus [129]	127	23	-	0.2	96.6	3.2	17.9	99.2	0.8	16.2	1.9	21.1	60.8	-	-	-	2.3
Cynara cornigera [120]	128	15	-	-	92.5	7.5	17.7	97.5	2.5	39.0	6.0	40.0	15.0	-	-	-	1.3
Cynara cardunculus [120]	129	12	-	-	90.6	9.4	17.7	98.1	1.9	46.8	5.7	33.4	14.1	-	-	-	1.2
Lactuca sativa [120]	130	18	-	-	95.6	4.4	17.8	99.0	1.0	20.6	3.4	15.9	60.1	-	-	-	2.2
Carthamus tinctorius [161]	131	8	-	37.7	55.4	9.6	14.9	102.7	-	65.2	7.0	8.9	21.6	-	-	-	0.9

Avicennia officinalis [135]	132	12	-	0.8	70.9	2.7	17.1	72.6	1.8	37.8	16.7	3.6	16.3	-	-	-	0.7
Acanthus ilicifolius [135]	133	12	-	40.1	54.8	0.4	14.7	93.5	1.8	77.5	4.8	7.8	5.2	-	-	-	0.4
Spathodea campanulata [134]	134	9	-	-	68.5	29.5	18.2	98.0	-	58.8	39.2	-	-	-	-	-	0.4
Aloysia citrodora [162]	135	10	-	-	94.0	5.5	17.4	89.9	9.6	40.9	18.9	15.7	24.0	-	-	-	1.2
Verbena officinalis [162]	136	11	-	-	97.2	2.8	17.6	98.5	1.5	30.5	15.8	17.7	36.0	-	-	-	1.6
Nepeta transcaucasica [163]	137	6	-	-	97.3	2.7	17.2	100.0	-	77.9	3.5	11.3	7.3	-	-	-	0.5
Lavandula X intermedia [164]	138	10	-	-	98.9	1.1	16.9	99.0	1.0	64.1	7.5	5.2	23.2	-	-	-	0.9
Thymbra capitata [165]	139	14	-	-	69.3	21.8	18.5	88.9	2.2	43.3	3.1	15.1	29.6	-	-	-	1.2
Thymus fallax [166]	140	10	-	0.1	89.3	1.2	17.5	90.2	0.4	27.9	0.3	19.5	42.9	-	-	-	1.7
Thymus kotschyanus var. glabrescens [166]	141	5	-	-	92.8	-	17.5	92.8	-	23.3	2.5	13.5	53.5	-	-	-	1.9
Thymus kotschyanus var. kotschyanus [166]	142	10	-	-	89.9	1.3	17.5	90.2	1.0	26.6	3.1	17.0	44.5	-	-	-	1.7
Thymus haussknechtii [166]	143	10	-	-	92.7	21.8	18.2	114.3	0.2	51.1	4.1	12.5	46.8	-	-	-	1.7
Thymus pubescens [166]	144	10	-	-	75.2	15.2	18.3	90.1	0.3	30.9	7.9	10.9	40.7	-	-	-	1.5
Vitex altissima [167]	145	7	-	0.1	99.9	-	17.3	100.0	-	39.5	13.9	6.9	39.7	-	-	-	1.5
Vitex negundo [167]	146	7	-	5.5	94.5	-	16.8	100.0	-	60.1	14.1	13.0	12.8	-	-	-	0.8
Vitex trifolia [167]	147	7	-	0.1	99.9	-	17.2	100.0	-	44.4	9.7	6.7	39.2	-	-	-	1.4
Scutellaria orientalis [163]	148	7	-	-	97.3	2.7	17.6	97.9	2.1	44.1	4.4	10.4	41.1	-	-	-	1.5
Satureja boissieri [166]	149	4	-	-	89.2	-	17.5	89.2	-	22.9	2.5	10.9	52.9	-	-	-	1.8
Satureja macrantha [163]	150	7	-	-	93.4	5.6	16.9	99.0	-	89.7	1.6	5.7	2.0	-	-	-	0.2
Satureja hortensis [164]	151	15	-	0.1	99.1	0.8	17.8	99.6	0.5	16.3	8.9	23.1	51.8	-	-	-	2.1
Prasium majus [120]	152	16	-	-	85.0	15.0	18.1	95.1	4.9	37.3	15.1	14.1	33.5	-	-	-	1.4
Origanum acutidens [166]	153	11	-	0.1	91.3	1.6	17.5	92.2	0.8	28.9	8.7	tr.	55.4	-	-	-	1.8
Origanum vulgare [166]	154	10	-	-	90.0	0.7	17.6	90.1	0.6	20.2	1.8	15.8	52.9	-	-	-	1.9
Ocimum basilicum [168]	155	16	-	0.2	98.9	0.9	17.2	99.6	0.4	43.1	10.1	12.1	34.7	-	-	-	1.4
Salvia euphratica [163]	156	5	-	-	100.0	-	17.3	100.0	-	73.7	2.2	20.1	4.0	-	-	-	0.6
Salvia officinalis [169]	157	8	-	-	83.5	16.6	18.1	100.1	-	19.7	20.6	14.0	45.8	-	-	-	1.9
Mentha cervina [170]	158	18	0.4	2.5	92.0	4.7	17.5	98.7	0.9	34.1	9.5	10.4	45.6	-	-	-	1.8
Mentha piperita [144]	159	14	-	2.3	91.1	6.6	17.6	97.7	2.3	35.8	6.6	32.0	25.6	-	-	-	1.5
Plantago lanceolata [122]	160	8	-	0.2	96.9	tr.	17.2	97.1	-	40.2	9.4	20.8	26.7	-	-	-	1.3
Antirrhinum sp. [122]	161	8	-	tr.	95.9	tr.	17.3	95.9	-	39.9	28.0	8.0	20.1	-	-	-	1.0
Olea europaea [171]	162	8	-	2.8	94.4	4.2	17.4	101.4	-	34.1	29.2	5.8	32.3	-	-	-	1.4
Solanum erianthum [127]	163	23	-	-	99.7	0.3	17.5	100.0	-	29.2	2.3	17.1	51.4	tr.	tr.	0.1	1.9
Solanum macrocarpon [125]	164	12	-	-	95.5	2.7	17.6	98.2	-	29.8	2.4	21.4	44.6	-	-	-	1.8
Solanum nigrum [120]	165	14	-	-	85.2	14.8	17.8	94.5	5.5	55.6	7.0	18.1	19.3	-	-	-	1.0
Nicotiana suaveolens [31]	166	7	-	-	100.0	-	17.4	100.0	-	19.5	7.5	8.6	64.4	-	-	-	2.2
Nicotiana arentsii [31]	167	7	-	-	100.0	-	17.5	100.0	-	16.5	8.7	11.8	63.0	-	-	-	2.2
Nicotiana wigandioides [31]	168	7	-	-	100.0	-	17.5	100.0	-	17.8	9.0	12.7	60.5	-	-	-	2.2
Nicotiana pauciflora [31]	169	7	-	-	100.0	-	17.6	100.0	-	16.2	5.9	17.1	60.8	-	-	-	2.2
Nicotiana setchellii [31]	170	7	-	-	100.0	-	17.5	100.0	-	18.3	7.6	9.3	64.8	-	-	-	2.2
Nicotiana raimondii [31]	171	7	-	-	100.0	-	17.4	100.0	-	17.4	9.1	10.2	63.3	-	-	-	2.2
Nicotiana rotundifolia [31]	172	7	-	-	100.0	-	17.4	100.0	-	18.7	7.9	10.3	63.1	-	-	-	2.2
Nicotiana maritima [31]	173	7	-	-	100.0	-	17.5	100.0	-	21.8	7	9.7	61.5	-	-	-	2.1
Nicotiana rosulata [31]	174	7	-	-	100.0	-	17.4	100.0	-	21.3	6.8	7.3	64.6	-	-	-	2.2
Nicotiana rosulata subsp. Ingulba [31]	175	7	-	-	100.0	-	17.4	100.0	-	21.5	7.6	7.7	63.2	-	-	-	2.1
Nicotiana occidentalis [31]	176	7	-	-	100.0	-	17.5	100.0	-	20.5	7.0	12.2	60.3	-	-	-	2.1
Nicotiana occidentalis subsp. Hesperis [31]	177	7	-	-	100.0	-	17.5	100.0	-	19.9	7.5	12.2	60.4	-	-	-	2.1
Nicotiana goodspeedii [31]	178	7	-	-	99.6	-	17.5	99.6	-	21.9	6.6	18.4	52.7	-	-	-	2.0
Nicotiana africana [31]	179	7	-	-	100.0	-	17.4	100.0	-	19.6	10.7	11.1	58.6	-	-	-	2.1
Nicotiana corymbosa [31]	180	7	-	-	100.0	-	17.6	100.0	-	17.2	3.7	14.1	65	-	-	-	2.3
Nicotiana solanifolia [31]	181	7	-	-	100.0	-	17.4	100.0	-	18.3	10.7	10.9	60.1	-	-	-	2.1
Nicotiana undulata [31]	182	7	-	-	100.0	-	17.5	100.0	-	16.1	9.3	12.0	62.6	-	-	-	2.2
Nicotiana umbratica [31]	183	7	-	-	100.0	-	17.5	100.0	-	20.5	7.4	8.1	64	-	-	-	2.2
Nicotiana trigonophylla [31]	184	7	-	-	100.0	-	17.5	100.0	-	19.7	7.3	11.1	61.9	-	-	-	2.2
Nicotiana stocktonii [31]	185	7	-	-	100.0	-	17.5	100.0	-	20.7	6.3	15.4	57.6	-	-	-	2.1
Nicotiana simulans [31]	186	7	-	-	100.0	-	17.5	100.0	-	21.7	7.1	10.9	60.3	-	-	-	2.1
Nicotiana noctiflora [31]	187	7	-	-	100.0	-	17.4	100.0	-	19.3	7.4	13.1	60.2	-	-	-	2.1
Nicotiana miersii [31]	188	7	-	-	100.0	-	17.5	100.0	-	17.0	7.7	14.1	61.2	-	-	-	2.2
Nicotiana megalosiphon [31]	189	7	-	-	100.0	-	17.4	100.0	-	21.8	7.3	10.2	60.7	-	-	-	2.1
Nicotiana longiflora [31]	190	7	-	-	100.0	-	17.4	100.0	-	20.5	6.0	13.4	60.1	-	-	-	2.1
Nicotiana langsdorffii [31]	191	7	-	-	100.0	-	17.5	100.0	-	18.00	7.2	10.1	64.7	-	-	-	2.2
Nicotiana knightiana [31]	192	7	-	-	100.0	-	17.4	100.0	-	18.5	9.7	8.4	63.4	-	-	-	2.2
Nicotiana gossei [31]	193	7	-	-	100.0	-	17.5	100.0	-	22.1	6.4	10.2	61.3	-	-	-	2.1
Nicotiana exigua [31]	194	7	-	-	100.0	-	17.4	100.0	-	17.6	8.4	11.4	62.6	-	-	-	2.2
Nicotiana bonariensis [31]	195	7	-	-	100.0	-	17.5	100.0	-	18.1	5.0	11.7	65.2	-	-	-	2.2
Nicotiana amplexicaulis [31]	196	7	-	-	100.0	-	17.5	100.0	-	19.8	8.0	5.0	67.2	-	-	-	2.2
Nicotiana cavicola [31]	197	7	-	-	100.0	-	17.4	100.0	-	19.6	7.1	9.0	64.3	-	-	-	2.2
Nicotiana clevelandii [31]	198	7	-	-	100.0	-	17.4	100.0	-	20.5	8.5	13.0	58.0	-	-	-	2.1
Nicotiana repanda [31]	199	7	-	-	100.0	-	17.5	100.0	-	19.4	7.2	15.1	58.3	-	-	-	2.1
Nicotiana paniculata [31]	200	7	-	-	100.0	-	17.4	100.0	-	19.8	9.8	8.0	62.4	-	-	-	2.1

Nicotiana excelsior [31]	201	7	-	-	100.0	-	17.4	100.0	-	21.0	8.7	6.3	64.0	-	-	-	2.1
Nicotiana tomentosa [31]	202	7	-	-	100.0	-	17.5	100.0	-	20.8	6.9	10.7	61.6	-	-	-	2.1
Nicotiana kawakamii [31]	203	7	-	-	103.0	-	17.4	100.0	-	21.4	8.8	11.6	61.2	-	-	-	2.2
Nicotiana nudicaulis [31]	204	7	-	-	100.0	-	17.4	100.0	-	19.2	8.4	12.7	59.7	-	-	-	2.1
Nicotiana attenuata [31]	205	7	-	-	100.0	-	17.5	100.0	-	17.6	7.4	13.7	61.3	-	-	-	2.2
Nicotiana glutinosa [31]	206	7	-	-	100.0	-	17.4	100.0	-	17.9	8.3	8.9	64.9	-	-	-	2.2
Nicotiana benthamiana [31]	207	7	-	-	100.0	-	17.4	100.0	-	19.2	9.6	5.7	65.5	-	-	-	2.2
Nicotiana tomentosiformis [31]	208	7	-	-	100.0	-	17.5	100.0	-	17.7	6.3	10.7	65.3	-	-	-	2.2
Nicotiana tabacum [172]	209	15	-	2.5	96.9	0.6	17.0	97.1	2.9	30.2	7.2	14.0	48.6	-	-	-	1.8
Nicotiana sylvestris [31]	210	7	-	-	100.0	-	17.5	100.0	-	17.8	8.4	10.6	63.2	-	-	-	2.2
Nicotiana rustica [31]	211	7	-	-	100.0	-	17.4	100.0	-	19.0	8.3	8.6	64.1	-	-	-	2.2
Nicotiana plumbaginifolia [31]	212	7	-	-	100.0	-	17.4	100.0	-	17.7	9.0	15.2	58.1	-	-	-	2.1
Nicotiana otophora [31]	213	7	-	-	100.0	-	17.6	100.0	-	17.4	4.0	10.0	68.6	-	-	-	2.3
Nicotiana glauca [31]	214	7	-	-	100.0	-	17.4	100.0	-	16.5	8.6	17.3	57.6	-	-	-	2.2
Nicotiana debneyi [31]	215	7	-	-	100.0	-	17.4	100.0	-	20.0	8.0	8.7	63.3	-	-	-	2.2
Nicotiana quadrivalvis var. bigelovii [31]	216	7	-	-	100.0	-	17.5	100.0	-	17.2	8.0	11.4	63.4	-	-	-	2.2
Nicotiana alata [31]	217	7	-	-	100.0	-	17.5	100.0	-	18.4	7.1	12.2	62.3	-	-	-	2.2
Nicotiana acuminata [31]	218	7	-	-	100.0	-	17.5	100.0	-	17.7	7.9	13.5	60.9	-	-	-	2.2
Capsicum annuum [173]	219	8	-	2.4	96.4	-	17.2	98.8	-	32.4	9.3	27.2	29.9	-	-	-	1.5
Hedera helix [122]	220	8	-	4.1	81.8	2.6	17.3	88.5	-	30.2	20.0	20.1	18.3	-	-	-	1.2
Anethum foeniculum [120]	221	17	-	-	95.5	4.6	17.5	97.0	3.1	33.2	5.1	29.0	32.8	-	-	-	1.6
Helosciadium nodiflorum [129]	222	16	-	1.0	92.5	6.3	17.7	99.2	0.6	23.7	5.4	24.6	46.1	-	-	-	1.9
Centella asiatica [174]	223	6	-	13.7	86.3	-	16.0	100.0	-	78.5	tr.	17.5	4.0	-	-	-	0.5
Crithmum maritimum [175]	224	8	-	-	100.6	-	17.3	100.6	-	29.2	7.8	30.1	33.5	-	-	-	1.7
Coriandrum sativum [176]	225	9	-	-	35.6	-	17.5	30.1	5.5	6.0	7.1	5.2	14.7	2.6	-	-	0.7
Apium graveolens [120]	226	14	-	-	87.4	18.4	18.1	104.9	0.9	39.5	14.6	24.6	27.1	-	-	-	1.5
Petroselinum crispum [120]	227	15	-	-	94.4	5.7	17.6	97.6	2.5	40.1	13.7	22.0	24.3	-	-	-	1.3
Daucus carota [120]	228	14	-	-	93.3	6.7	17.7	99.2	0.8	32.2	14.0	25.7	28.1	-	-	-	1.5
Salix sp. [122]	229	8	-	3.4	86.9	2.9	17.3	93.2	-	39.5	22.4	9.6	21.7	-	-	-	1.1
Ceriops decandra [135]	230	13	-	25.7	71.9	0.8	15.2	96.8	1.6	81.0	9.5	4.2	3.7	-	-	-	0.3
Bruguiera cylindrica [135]	231	14	-	12.8	82.8	0.9	15.7	93.9	2.6	81.6	9.6	3.0	2.3	-	-	-	0.2
Rhizophora apiculata [135]	232	12	-	19.3	78.9	0.2	15.6	97.3	1.1	78.1	5.0	7	8.3	-	-	-	0.4
Rhizophora mucronata [135]	233	12	-	12.5	85.1	0.4	15.9	96.4	1.6	77.1	7.7	4.3	8.9	-	-	-	0.4
Cnidoscolus aconitifolius [177]	234	20	-	0.6	97.3	1.8	17.5	98.8	0.9	30.8	9.9	9.0	50.0	-	-	-	1.8
Croton zambesicus [178]	235	12	-	1.0	95.7	3.2	17.3	99.9	-	41.8	36.9	18.3	2.9	-	-	-	0.8
Excoecaria agallocha [179]	236	13	-	18.9	78.0	0.9	15.5	93.4	3.7	85.6	1.6	3.1	6.8	-	-	-	0.3
Euphorbia lathyris [180]	237	6	-	-	69.6	-	17.5	69.6	-	16.9	1.9	12.5	38.3	-	-	-	1.4
Euphorbia lagascae [180]	238	7	-	-	62.1	0.4	17.5	62.5	-	14.2	1.0	9.3	38.0	-	-	-	1.3
Linum usitatissimum [181]	239	9	-	0.1	96.5	0.9	17.1	97.5	-	35.8	15.3	3.5	42.9	-	-	-	1.5
Melilotus siculus [143]	240	4	-	0.5	91.0	-	16.7	91.5	-	33.5	58.0	-	-	-	-	-	0.6
Senna sophera [182]	241	22	1.0	6.4	45.4	46.3	18.0	96.9	2.2	71.4	8.3	10.2	8.8	0.4	-	-	0.6
Senna tora [183]	242	21	-	0.9	68.8	12.6	18.3	73.2	9.1	40.2	12.7	13.2	16.1	-	-	-	0.9
Senna siamea [134]	243	6	-	-	106.4	5.0	17.0	111.4	-	76.2	19.4	15.8	-	-	-	-	0.5
Delonix regia [134]	244	8	-	4.7	83.5	12.5	17.3	100.7	-	67.8	18.2	14.7	-	-	-	-	0.5
Trifolium angustifolium [139]	245	22	2.0	3.3	85.7	9.0	17.4	98.7	1.3	38.2	6.8	20.3	34.7	-	-	-	1.5
Trifolium alexandrinum [143]	246	6	-	-	94.6	-	16.5	94.6	-	48.4	8.7	7.6	29.9	-	-	-	1.1
Trifolium pratense [122]	247	8	-	tr.	98.7	tr.	17.2	98.7	-	41.8	10.5	5.0	41.4	-	-	-	1.5
Cassia fistula [134]	248	5	-	-	95.1	2.8	17.2	97.9	-	56.6		41.3	-	-	-	-	0.8
Ceratonia siliqua [132]	249	21	-	6.4	92.7	1.0	16.4	96.8	3.3	69.4	4.9	2.8	23.0	-	-	-	0.8
Vigna unguiculata [125]	250	12	-	-	97.7	2.7	17.4	100.0	0.4	39.6	3.2	7.2	50.4	-	-	-	1.7
Vicia faba [120]	251	12	-	-	93.5	9.5	17.8	101.8	1.2	27.5	9.2	52.1	14.2	-	-	-	1.6
Onobrychis viciifolia [122]	252	8	-	tr.	98.0	tr.	17.2	98.0		41.6	9.3	14.3	32.8	-	-	-	1.4
Lathyrus ochrus [120]	253	16	-	-	96.1	3.9	17.7	96.6	3.4	30.9	4.6	13.9	50.6	-	-	-	1.8
Moringa oleifera [184]	254	15	-	1.0	94.7	4.2	17.6	99.9	-	27.9	6.7	11.0	54.3	-	-	-	1.9
Arabidopsis halleri [185]	255	24	-	tr.	98.9	1.1	17.4	99.8	0.2	22.1	7.4	21.5	49.0	-	-	-	2.0
Arabidopsis lyrata [185]	256	19	-	0.1	98	1.9	17.2	99.1	0.9	37.5	8.7	20.6	33.2	-	-	-	1.5
Sinapis arvensis [143]	257	10	-	1.1	87	1.6	16.6	89.7	-	48.4	6.4	6.3	28.6	-	-	-	1.1
Eruca vesicaria subsp. Sativa [120]	258	17	-	-	90.4	9.6	17.6	94.4	5.6	41.6	8.9	10.3	39.2	-	-	-	1.5
Brassica napus [186]	259	13	-	0.2	90.4	4.5	17.7	95.1	-	25.9	5.8	7.3	56.1	-	-	-	1.9
Cucurbita pepo [187]	260	11	-	0.5	92.2	5.8	17.5	98.1	0.4	44.0	7.5	5.1	41.9	-	-	-	1.4
Cucurbita pepo subsp. pepo [187]	261	13	-	-	81.6	15.6	18.2	97.2	-	36.7	11.1	16.2	33.2	-	-	-	1.4
Cucurbita pepo var. melopepo [187]	262	11	-	-	89.6	7.2	17.8	96.8	-	27.9	8.8	27.6	32.5	-	-	-	1.6
Bryonia cretica [120]	263	17	-	-	95.6	5.9	17.6	99.0	2.4	32.7	9.4	33.6	25.7	-	-	-	1.5
Bryonia dioica [129]	264	20	tr.	0.2	96.2	3.6	17.8	99.3	0.7	24.1	1.6	6.5	67.8	-	-	-	2.2
Montia fontana [129]	265	22	-	0.3	86.5	13.2	18.0	99.2	0.8	25.6	7.5	18.2	48.7	-	-	-	1.9
Sesuvium portulacastrum [188]	266	12	-	2.2	91.3	2.7	16.9	95.6	0.6	50.2	21.2	10.6	14.2	-	-	-	0.9
Amaranthus cruentus [125]	267	11	-	-	98.2	1.6	17.5	100.0	-	28.3	4.4	17.2	50.1	-	-	-	1.9
Portulaca oleracea [189]	268	9	-	-	96.5	3.5	17.8	100.0	-	19.9	5.5	12.1	62.5	-	-	-	2.2
Silene vulgaris [120]	269	15	-	-	95.7	4.3	17.6	98.1	1.9	29.7	3.2	26.8	39.9	0.4	-	-	1.8

Rumex pulcher [129]	270	26	tr.	0.4	96.6	3.0	17.9	99.2	0.8	14.9	4.8	17.2	63.0	-	0.1	-	2.3
Rumex papillaris [129]	271	24	tr.	0.1	96.8	3.1	17.7	98.9	1.1	16.1	9.1	23.1	51.7	-	-	-	2.1
Rumex patientia [190]	272	26	7.6	0.2	44.5	47.7	17.7	98.8	1.2	65.5	6.6	14.2	12.3	-	0.4	1.0	0.8
Rumex obtusifolius [120]	273	18	-	-	94.8	6.3	17.7	99.6	1.5	30.5	6.3	21.8	42.5	-	-	-	1.8
Salicornia europaea [16]	274	13	-	-	91.6	8.6	17.8	95.4	4.8	25.4	10.1	23.3	41.4	-	-	-	1.8
Salicornia brachiata [191]	275	14	-	63.2	36.0	tr.	13.7	97.9	1.3	84.3	tr.	10.6	4.3	-	-	-	0.3
Beta vulgaris [120]	276	15	-	-	97.3	5.8	17.6	100.0	3.1	28.0	11.2	25.0	38.9	-	-	-	1.8
Suaeda monoica [191]	277	13	-	11.0	83.2	4.8	16.5	97.4	1.6	63.1	5.3	22.2	8.4	-	-	-	0.7
Suaeda altissima [192]	278	24	-	0.1	96.0	3.9	17.7	99.3	0.7	29.6	9.1	22.2	39.1	-	-	-	1.7
Suaeda maritima [191]	279	13	-	24.3	70.3	4.0	15.7	96.9	1.7	74.2	3.9	14.3	6.2	-	-	-	0.5
Spinacia oleracea [120]	280	18	-	-	89.9	13.8	18.1	102.5	1.2	24.2	23.9	14.4	41.1	-	-	-	1.8
Chenopodium album [193]	281	15	-	tr.	99.4	0.6	17.5	99.9	0.1	21.5	5.2	14.3	59.0	-	-	-	2.1
Hippophae rhamnoides [137]	282	13	-	3.4	96.6	tr.	16.1	100.0	-	35.1	54.3	10.6	tr.	tr.	-	-	0.8
Rubus ulmifolius [139]	283	22	0.3	0.8	76.9	22.0	18.5	98.6	1.4	39.7	4.6	16.1	39.6	-	-	-	1.6
Rosa elliptica subsp. *inodora* [194]	284	17	-	-	97.7	1.3	17.9	98.9	0.1	6.7	19.2	50.3	22.8	-	-	-	1.9
Rosa dumalis [194]	285	17	-	-	97.9	1.4	17.9	99.2	0.1	5.7	15.6	48.5	29.5	-	-	-	2.0
Rosa villosa [194]	286	15	-	-	98.0	1.6	17.9	99.5	0.1	5.8	19.9	44.4	29.5	-	-	-	2.0
Rosa subcanina [194]	287	17	-	-	98.1	1.5	18.0	99.5	0.1	6.0	15.0	49.9	28.7	-	-	-	2.0
Rosa micrantha [195]	288	19	0.1	1.6	76.7	21.6	18.5	88.6	11.4	43.2	3.3	21.2	32.33	-	-	-	1.4
Rosa rugosa [194]	289	17	-	-	97.6	1.9	18.0	99.4	0.1	4.9	16.2	50.5	27.9	-	-	-	2.0
Rosa rubiginosa [194]	290	17	-	-	98.1	1.5	18.0	99.5	0.1	5.5	15.4	47.3	31.4	-	-	-	2.0
Rosa canina [194]	291	17	-	-	97.8	1.5	17.9	99.2	0.1	7.2	21.3	51.7	19.1	-	-	-	1.8
Rosa majalis [144]	292	13	-	-	96.7	3.5	17.5	98.9	1.3	34.9	5.9	20.2	39.1	-	-	-	1.6
Malus orientalis [196]	293	15	-	-	84.4	15.6	18.0	98.9	1.1	52.1	14.4	24.7	8.8	-	-	-	0.9
Malus domestica [197]	294	21	-	0.5	98.2	1.3	17.5	99.1	0.9	35.2	27.5	31.1	6.2	-	-	-	1.1
Mespilus germanica [47]	295	21	-	0.9	93.3	5.8	17.6	98.9	1.1	40.5	9.5	22.6	27.4	-	-	-	1.4
Cydonia oblonga [198]	296	22	-	0.8	96.8	2.0	17.5	98.6	1.0	32.3	12.9	26.6	27.8	-	-	-	1.5
Pyrus communis [17]	297	16	-	0.9	95.9	3.2	17.3	99.3	0.7	45.0	4.7	8.00	42.3	-	-	-	1.5
Pyrus caucasica [17]	298	16	-	0.6	87.4	12.0	17.6	99.4	0.6	50.6	8.8	8.2	32.4	-	-	-	1.2
Eriobotrya japonica [132]	299	22	-	4.4	94.3	1.4	17.1	97.8	2.3	37.2	10.3	19.3	33.3	-	-	-	1.5
Prunus spinosa [199]	300	35	0.2	1.7	69.3	11.3	17.4	81.4	1.1	47.1	28.6	3.1	2.6	tr.	1.1	tr.	0.5
Prunus dulcis [200]	301	11	-	0.7	94.3	4.6	17.6	99.6	-	33.0	10.4	33.5	22.6	-	-	-	1.5
Urtica dioica [201]	302	20	-	1.3	72.8	25.4	18.3	96.7	2.8	47.2	6.9	22.3	22.6	0.5	-	-	1.2
Morus nigra [202]	303	13	-	-	92.2	7.8	17.6	97.6	2.4	42.6	3.8	16.1	37.5	-	-	-	1.5
Morus alba [132]	304	24	-	15.2	84.7	0.2	16.5	97.5	3.6	34.9	12.5	23.4	29.3	tr.	-	-	1.5
Ficus carica [132]	305	24	-	2.1	96.0	1.9	17.2	94.2	5.8	43.9	5.3	11.5	39.3	tr.	-	-	1.5
Humulus lupulus [129]	306	20	tr.	0.1	92.7	7.0	17.8	98.9	0.9	28.7	2.5	30.1	38.5	-	-	-	1.8
Persea americana [132]	307	24	-	5.6	89.3	5.1	16.9	92.9	7.1	47.5	9.2	18.8	24.5	tr.	-	-	1.2
Pistacia lentiscus [203]	308	20	-	5.1	83.4	2.4	17.2	88.8	2.1	21.9	3.7	16.4	48.9	-	tr.	-	1.8
Azadirachta indica [204]	309	20	-	2.2	78.6	19.1	17.7	98.4	1.5	45.1	10.1	24.2	13.1	7.4	-	-	1.3
Citrus limon [132]	310	22	-	6.0	91.9	2.1	16.5	96.3	3.7	63.6	6.5	13.7	16.2	-	-	-	0.8
Ephedra chilensis [205]	311	20	-	-	87.0	10.9	17.9	96.9	1.0	21.4	7.5	32.5	33.6	2.9	-	-	1.9
Ephedra equisetina [205]	312	21	-	0.4	86.2	13.1	17.9	98.8	0.9	22.4	5.9	24.4	42.3	4.7	-	-	2.0
Ephedra fragilis [205]	313	17	-	0.5	92.8	6.1	17.7	98.0	1.4	24.6	10.8	18.7	43.8	1.5	-	-	1.9
Ephedra gerardiana [205]	314	20	-	-	87.4	12.5	17.9	99.3	0.6	26.2	7.9	20.9	42.1	2.8	-	-	1.9
Ephedra distachya [205]	315	17	-	0.5	90.0	8.9	17.7	99.0	0.4	28.8	8.8	28.9	32.4	0.5	-	-	1.7
Gnetum gnemon [205]	316	18	-	1.1	73.0	26.1	18.1	98.7	1.5	34.0	9.1	4.6	36.1	16.4	-	-	1.9
Welwitschia mirabilis [205]	317	16	-	0.4	96.3	2.5	17.4	97.7	1.5	30.0	14.0	9.1	46.1	-	-	-	1.7
Ginkgo biloba [206]	318	14	1.0	-	85.7	13.5	17.8	94.9	5.3	29.2	11.8	12.1	45.1	2	-	-	1.8
Ceratozamia robusta [205]	319	20	-	0.4	94.0	5.5	17.5	96.6	3.3	26.9	12.2	21	38.4	1.4	-	-	1.8
Lepidozamia hopei [205]	320	20	-	0.5	88.3	10.6	17.7	97.7	1.7	26.6	8.3	15.3	44.2	5.0	-	-	1.9
Macrozamia riedlei [207]	321	9	-	0.5	92.3	0.9	17.3	93.7	-	36.9	29.4	15.2	12.2	-	-	-	1.0
Macrozamia moorei [205]	322	19	-	0.3	90.5	9.2	17.7	98.2	1.8	25.6	7.8	21.0	40.8	4.8	-	-	1.9
Microcycas calocoma [205]	323	23	-	0.8	89.7	9.2	17.7	99.7	tr.	27.5	11.5	7	50.2	3.5	-	-	1.9
Encephalartos lebomboensis [205]	324	19	-	0.8	80.7	18.8	17.9	98.6	1.7	38.5	13.1	12.5	30.8	5.4	-	-	1.5
Dioon edule [205]	325	21	-	0.6	88.8	11.3	17.7	98.5	2.2	30.0	9.2	18.4	36.8	6.3	-	-	1.8
Zamia furfuracea [208]	326	21	-	0.2	79.8	20.1	18.0	98.3	1.8	30.4	10.1	8.2	44.7	6.7	-	-	1.9
Stangeria eriopus [205]	327	17	-	1.9	89.2	8.6	17.4	97.6	2.1	37.0	11.7	12.5	36.6	1.9	-	-	1.5
Bowenia spectabilis [205]	328	16	-	2.2	88.3	9.5	17.6	96.6	3.4	32.0	9.4	19.8	38.8	-	-	-	1.7
Cycas armstrongii [205]	329	16	-	-	98.0	2.0	17.4	97.5	2.5	29.0	10.0	14.9	45.9	0.2	-	-	1.8
Cycas siamensis [205]	330	20	-	-	97.8	2.1	17.4	97.3	2.6	27.7	13.0	15.6	42.8	0.8	-	-	1.8
Cycas revoluta [209]	331	24	-	0.6	97.2	2.3	17.3	96.6	3.5	34.2	9.7	31.0	25.2	-	-	-	1.5
Pectinopitys ferruginea [205]	332	17	-	0.5	91.4	8.1	17.5	98.9	1.1	29.4	8.5	22.6	37.4	2.1	-	-	1.7
Podocarpus salignus [205]	333	21	-	2.3	89.1	10.7	17.4	100.8	1.3	30.7	8.7	15.9	43.9	2.9	-	-	1.8
Podocarpus nivalis [205]	334	18	-	0.5	83.9	15.6	17.7	84.0	16.0	38.9	3.9	17.6	29.9	9.7	-	-	1.7
Podocarpus macrophyllus [205]	335	14	-	-	80.7	17.9	17.7	94.7	3.9	34.4	4.8	11.5	43.8	4.1	-	-	1.8
Podocarpus macrophyllus var. maki [205]	336	17	-	0.9	77.1	18.0	17.7	95.2	0.8	35.2	11.1	7.3	39.0	3.4	-	-	1.5
Prumnopitys andina [205]	337	17	-	1.6	81.8	15.0	17.7	96.9	1.5	32.9	7.4	12.7	41.9	3.5	-	-	1.7

Prumnopitys taxifolia [205]	338	18	-	-	88.0	9.2	17.4	80.2	17.0	34.4	4.5	14.9	38.8	4.6	-	-	1.7
Pherosphaera fitzgeraldii [205]	339	21	-	0.2	79.9	17.4	17.6	90.2	7.3	30.3	20.1	15.6	26.0	5.5	-	-	1.5
Dacrydium cupressinum [205]	340	19	-	1.8	80.4	18.1	17.8	99.7	0.6	34.4	6.7	12.5	41.5	5.2	-	-	1.8
Agathis robusta [205]	341	18	-	2.5	84.0	3.6	17.3	88.7	1.4	27.5	11.3	6.4	44.2	0.7	-	-	1.6
Agathis moorei [205]	342	16	-	0.4	84.7	9.9	17.6	94.0	1.0	31.2	8.9	8.5	43.9	2.5	-	-	1.7
Agathis australis [205]	343	17	-	3.1	60.8	36.2	18.2	99.1	1.0	27.8	10.9	22.3	33.0	6.1	-	-	1.8
Araucaria montana [205]	344	20	-	0.2	74.5	24.5	17.9	63.5	35.7	47.5	22.3	6.9	13.1	9.4	-	-	1.1
Araucaria luxurians [205]	345	17	-	0.3	88.4	11.6	17.5	88.1	12.2	42.1	12.0	11.0	33.7	1.5	-	-	1.4
Araucaria cunninghamii [205]	346	19	-	0.3	70.2	19.6	17.3	60.6	29.5	41.0	6.9	16.1	16.7	9.4	-	-	1.3
Araucaria angustifolia [205]	347	19	-	tr.	72.9	26.9	18.1	98.6	1.2	23.8	5.7	19.5	47.5	3.3	-	-	2.0
Araucaria araucana [205]	348	19	-	1.2	83.0	15.5	17.5	98.0	1.7	22.1	25.9	13.7	35.3	2.7	-	-	1.7
Keteleeria evelyniana [205]	349	17	-	1.1	70.6	28.4	18.4	98.0	2.1	43.4	11.1	7.6	34.9	3.1	-	-	1.4
Abies vejarii [205]	350	22	-	5.9	74.6	19.2	17.7	95.0	4.7	39.0	9.3	13.2	25.7	12.5	-	-	1.6
Abies nordmanniana [208]	351	25	-	1.0	90.4	8.6	17.7	94.1	5.9	21.1	8.3	13.6	52.3	4.7	-	-	2.1
Abies procera [208]	352	25	-	0.1	93.7	6.2	17.7	94.7	5.3	24.2	12.4	17.9	40.3	5.2	-	-	1.9
Abies amabilis [208]	353	25	-	0.6	90.9	8.5	17.8	94.4	5.6	20.6	7.8	14.9	50.3	6.4	-	-	2.1
Abies concolor [205]	354	22	-	2.3	75.9	21.6	18.2	95.4	4.4	33.8	11.6	10.0	39.0	5.4	-	-	1.7
Abies sachalinensis [205]	355	22	-	9.6	73.7	16.9	17.3	92.7	7.5	48.9	12.6	15.3	18.6	4.8	-	-	1.2
Abies veitchii [205]	356	21	-	1.3	72.6	19.2	17.9	86.2	6.9	38.5	9.9	18.9	21.0	4.8	-	-	1.3
Abies cephalonica [205]	357	21	-	5.5	58.1	28.5	18.5	87.7	4.4	38.1	6.5	8.9	32.8	5.8	-	-	1.5
Abies pinsapo [205]	358	20	-	0.4	68.0	31.5	18.7	96.8	3.1	43.5	12.8	9.5	30.5	3.6	-	-	1.4
Abies grandis [205]	359	22	-	1.0	64.0	24.4	18.5	82.9	6.5	27.8	10.6	11.7	31.9	7.4	-	-	1.6
Abies alba [205]	360	20	-	1.2	73.1	25.7	18.3	94.5	5.5	40.6	13.8	11	29.8	4.8	-	-	1.4
Tsuga canadensis [208]	361	25	-	0.3	86.4	13.3	17.9	98.1	1.9	21.2	4.0	11.9	59.0	3.9	-	-	2.2
Tsuga heterophylla [208]	362	25	-	0.2	91.7	8.1	17.7	96.8	3.2	24.8	6.3	9.6	55.0	4.3	-	-	2.1
Pseudotsuga sinensis var. wilsoniana [208]	363	9	-	-	99.5	0.5	17.5	100.0	-	29.3	53.4	16.4	0.9	-	-	-	0.9
Pseudotsuga macrocarpa [205]	364	21	-	1.8	74.0	24.1	18.2	97.7	2.2	34.5	5.3	9.0	43.8	7.3	-	-	1.8
Pseudotsuga menziesii [205]	365	21	-	3.8	57.9	38.0	18.6	96.5	3.2	49.1	4.1	13.4	25.4	7.7	-	-	1.4
Pseudolarix amabilis [205]	366	18	-	1.7	82.7	12.8	17.6	95.7	1.5	25.0	5.8	11.6	43.8	11.0	-	-	2.0
Pinus strobiformis [205]	367	22	-	4.8	66.6	27.5	18.0	97.3	1.6	42.3	5.9	11.1	34.3	5.3	-	-	1.5
Pinus monophylla [205]	368	22	-	4.3	75.2	18.5	17.9	97.5	0.5	31.0	11.6	17.2	35.2	3.0	-	-	1.6
Pinus koraiensis [210]	369	20	-	12.2	74.6	11.5	17.1	95.6	2.7	37.2	5.9	17.6	34.9	2.7	-	-	1.6
Pinus pinaster [205]	370	18	-	8.3	50.1	40.8	18.2	98.0	1.2	54.1	6.1	14.7	22.7	1.6	-	-	1.1
Pinus halepensis [205]	371	20	-	5.1	72.5	21.6	17.8	97.9	1.3	35.5	5.4	11.5	43.3	3.5	-	-	1.7
Pinus bungeana [205]	372	23	-	6.0	75.6	16.1	17.7	97.2	0.5	27.0	13.9	19.5	34.7	2.6	-	-	1.7
Pinus aristata [205]	373	22	-	2.5	72.0	25.3	18.1	98.2	1.3	36.8	5.8	15.2	38.1	3.6	-	-	1.7
Pinus nigra [208]	374	25	-	8.9	76.7	14.5	17.2	99.2	0.9	34.6	4.6	9.5	48.2	3.2	-	-	1.8
Pinus cembra [205]	375	22	-	2.5	72.1	25.4	18.0	95.6	4.4	37.8	5.2	12.2	38.4	6.4	-	-	1.7
Pinus ponderosa [207]	376	12	-	7.9	83.1	9.2	17.3	100.2	-	41.5	29.2	5.4	24.1	-	-	-	1.1
Pinus jeffreyi [205]	377	23	-	8.9	49.9	40.7	18.2	98.4	1.1	53.1	6.0	11.2	26.5	2.7	-	-	1.2
Pinus resinosa [205]	378	22	-	0.3	77.0	22.3	18.1	97.7	1.8	28.5	6.8	15.5	43.0	5.7	-	-	1.9
Pinus palustris [205]	379	20	-	3.1	62.5	30.0	17.9	94.0	1.6	44.2	3.0	12.8	33.1	2.5	-	-	1.4
Pinus sylvestris [208]	380	25	-	1.7	84.5	13.8	17.7	99.3	0.7	20.6	8.0	17.2	49.0	5.2	-	-	2.1
Pinus pinea [205]	381	18	-	5.8	76.2	17.5	17.4	89.8	9.7	42.6	5.901	10.3	39.3	1.4	-	-	1.5
Pinus wallichiana [205]	382	22	-	5.5	77.8	16.4	17.7	98.0	1.7	31.7	9.5	16.5	38.3	3.7	-	-	1.7
Pinus contorta [208]	383	25	-	2.4	91.1	6.5	17.7	99.6	0.4	18.2	18.3	22.9	37.9	2.7	-	-	1.9
Picea obovata [208]	384	25	-	1.2	88.5	10.3	17.7	95.9	4.1	22.7	6.3	15.8	49.0	6.2	-	-	2.1
Picea orientalis [208]	385	25	-	4.0	88.3	7.6	17.4	96.3	3.6	29.6	4.8	14.0	46.4	5.1	-	-	1.9
Picea asperata [205]	386	21	-	0.5	50.9	48.6	19.1	97.3	2.7	60.6	3.6	11.0	22.4	2.4	-	-	1.0
Picea chihuahuana [205]	387	20	-	0.3	76.3	23.2	18.3	97.5	2.3	32.7	8.5	15.1	39	4.5	-	-	1.7
Picea torano [205]	388	21	-	1.9	84.8	13.6	17.9	97.2	3.1	28.0	12.8	18.3	36.2	5	-	-	1.8
Picea omorica [205]	389	22	-	0.3	44.7	54	19.6	96.7	2.3	58.0	6.8	12.9	18.4	2.9	-	-	1.0
Picea mariana [208]	390	25	-	4.1	84.3	11.2	17.5	96.1	3.5	29.2	5.1	14.2	44.7	6.4	-	-	1.9
Picea engelmannii [208]	391	25	-	4.7	88.7	6.6	17.3	96.7	3.3	28.6	6.1	15.2	45.6	4.5	-	-	1.9
Picea sitchensis [208]	392	25	-	2.4	93.5	4.2	17.4	98.3	1.6	27.6	12.7	12.5	41.8	5.3	-	-	1.8
Picea pungens [210]	393	20	-	5.8	87.2	6.7	17.2	96.1	3.6	36.6	9.7	19.1	30.7	3.6	-	-	1.5
Picea glauca [205]	394	20	-	0.6	54.1	45.0	19.1	96.6	3.1	54.8	4.5	11.7	23.9	4.8	-	-	1.2
Picea abies [208]	395	25	-	1.4	92.9	5.7	17.6	97.6	2.4	23.1	6.1	12.6	51.5	6.7	-	-	2.1
Larix gmelinii [211]	396	21	-	0.9	87.5	11.7	17.9	97.6	2.5	44.1	12.5	6.7	36.8	-	-	-	1.4
Larix lyallii [205]	397	22	-	2.6	87.4	10.1	17.4	97.1	3.0	32.1	9.4	6.2	47.6	4.8	-	-	1.8
Larix decidua [208]	398	25	-	0.2	91.8	8.0	17.7	98.4	1.6	21.3	7.0	13.8	54.2	3.7	-	-	2.1
Larix sibirica [210]	399	20	-	0.2	93.3	6.9	17.5	98.0	2.4	31.7	5.9	8.9	49.9	4	-	-	1.9
Larix kaempferi [211]	400	21	-	1.5	87.7	11.0	17.9	98.4	1.8	38.4	9.3	6.6	45.9	0	-	-	1.6
Larix laricina [205]	401	20	-	0.9	90.0	9.1	17.5	96.5	3.5	29.5	6.3	8.2	49.7	6.3	-	-	2.0
Cedrus atlantica [205]	402	22	-	0.6	69.6	28.1	18.5	96.1	2.2	34.8	7.4	12.8	38.9	4.4	-	-	1.7
Cedrus libani [205]	403	20	-	0.5	53.1	45.6	19.3	97.6	1.6	55.3	7.5	7.1	27.7	1.6	-	-	1.1
Cedrus deodara [208]	404	25	-	5.2	87.3	7.5	17.3	97.5	2.5	27.5	4.61	10.8	52.6	4.5	-	-	2.0
Sciadopitys verticillata [205]	405	18	-	1.9	84.4	13.6	17.6	99.1	0.8	32.0	7.8	27.1	28.6	4.4	-	-	1.7

Torreya californica [205]	406	21	-	1.4	81.6	16.8	17.7	97.8	2.0	30.6	4.5	15.3	45.7	3.7	-	-	1.9
Cephalotaxus sinensis [205]	407	15	-	tr.	85.2	14.7	17.6	99.2	0.7	33.3	6.2	10.6	43.0	6.8	-	-	1.8
Cephalotaxus fortunei [205]	408	19	-	tr.	82.4	15.7	17.8	96.3	1.8	27.5	7.0	10.8	48.0	4.8	-	-	1.9
Cephalotaxus harringtonii [205]	409	21	-	tr.	86.0	13.3	17.8	97.9	1.4	25.5	8.1	11.2	49.9	4.6	-	-	2.0
Taxus cuspidata [205]	410	22	-	0.7	80.3	18.8	17.9	98.3	1.5	26.4	5.3	11.3	49.6	7.2	-	-	2.1
Taxus brevifolia [205]	411	22	-	0.1	87.6	12.5	17.7	99.2	1.0	20.5	4.1	7.6	64.4	3.6	-	-	2.3
Taxus baccata [205]	412	20	-	0.4	85.6	13.1	17.8	96.7	2.4	23.8	7.8	10.8	51.4	5.3	-	-	2.1
Callitris preissii [205]	413	19	-	5.1	79.1	15.7	17.6	98.3	1.6	38.7	3.9	11.0	43.8	2.5	-	-	1.7
Widdringtonia schwarzii [205]	414	17	-	3.1	69.5	27.4	18.2	97.7	2.3	40.5	8.3	9.6	38.2	3.4	-	-	1.6
Hesperocyparis bakeri [205]	415	23	-	1.4	78.1	19.7	17.8	97.4	1.8	26.5	5.0	19.3	43.8	4.6	-	-	1.9
Hesperocyparis lusitanica [205]	416	21	-	1.1	79.8	17.9	17.9	96.8	2.0	27.3	10.9	19.3	38.9	2.4	-	-	1.8
Hesperocyparis goveniana [205]	417	22	-	3.1	78.0	16.1	17.6	94.9	2.3	32.5	6.1	17.1	36.9	4.6	-	-	1.7
Austrocedrus chilensis [205]	418	19	-	3.3	67.8	28.4	17.9	97.3	2.2	48.7	3.5	13.8	29.9	3.6	-	-	1.4
Sequoiadendron giganteum [205]	419	20	-	2.8	74.6	22.0	17.9	98.6	0.8	32.1	4.0	15.7	43.9	3.7	-	-	1.8
Callitropsis nootkatensis [208]	420	22	-	0.5	85.1	14.7	17.8	99.8	0.5	25.9	4.4	23.9	43.2	2.9	-	-	1.9
Callitropsis funebris [205]	421	17	-	3.3	67.5	26.7	18.0	95.6	1.9	34.3	11.2	10.9	36.1	5.0	-	-	1.6
Platycladus orientalis [205]	422	19	-	4.8	71.7	23.1	17.8	96.7	2.9	37.0	4.3	12.8	41.5	4.0	-	-	1.7
Chamaecyparis thyoides [208]	423	22	-	0.6	77.3	22.1	18.0	99.0	1.0	21.8	2.8	13.1	54.6	7.7	-	-	2.2
Chamaecyparis pisifera [208]	424	22	-	0.3	86.3	13.4	17.9	99.2	0.8	21.9	2.1	15.9	57.3	2.8	-	-	2.2
Chamaecyparis hodginsii [205]	425	17	-	2.5	65.7	32.0	18.3	98.8	1.4	44.0	7.6	12.8	32.6	3.2	-	-	1.4
Chamaecyparis lawsoniana [208]	426	22	-	2.0	86.7	10.4	17.6	98.6	0.5	23.6	9.5	16.5	46.0	3.5	-	-	1.9
Taiwania cryptomerioides [205]	427	19	-	1.3	80.9	16.2	17.9	97.1	1.3	32.0	2.2	17.2	42.8	4.2	-	-	1.8
Juniperus sabina [205]	428	18	-	4.0	67.9	21.2	17.6	89.9	3.2	42.0	8.4	8.3	29.7	4.7	-	-	1.3
Juniperus communis [208]	429	22	-	0.5	85.4	4.1	17.4	89.1	0.9	22.9	4.1	4.1	57.5	1.4	-	-	1.9
Juniperus chinensis [205]	430	23	-	4.7	58.8	34.2	18.1	96.4	1.3	43.5	5.6	10.6	32.0	6.0	-	-	1.5
Juniperus virginiana [208]	431	22	-	3.9	76.6	19.5	17.7	99.3	0.7	32.1	4.3	14.5	44.8	4.3	-	-	1.9
Taxodium mucronatum [205]	432	16	-	0.2	83.2	15.3	17.8	97.7	1.0	23.3	5.6	11.3	53.8	4.7	-	-	2.1
Taxodium distichum var. distichum [205]	433	14	-	3.0	89.7	3.1	17.2	94.4	1.4	36.3	5.7	10.9	42.9	-	-	-	1.6
Taxodium distichum var. imbricatum [205]	434	14	-	0.6	87.9	11.4	17.7	98.9	1.0	31.5	4.7	15.2	46.1	2.4	-	-	1.8
Sequoia sempervirens [205]	435	16	-	11.0	69.4	19.6	17.4	99.1	0.9	42.8	2.5	9.9	43.1	1.7	-	-	1.6
Cunninghamia lanceolata [205]	436	16	-	0.9	86.3	12.6	17.7	98.4	1.4	22.4	3.3	9.1	60.8	4.2	-	-	2.2
Cunninghamia lanceolata var. konishii [205]	437	20	-	2.1	81.2	15.0	17.8	96.7	1.6	32.0	2.8	7.9	52.1	3.5	-	-	1.9
Athrotaxis laxifolia [205]	438	22	-	0.9	71.1	27.0	18.2	95.2	3.8	41.3	2.1	11.0	40.4	4.2	-	-	1.6
Thujopsis dolabrata [205]	439	20	-	1.9	67.9	29.5	18.2	93.8	5.5	44.1	8.7	10.1	33.5	2.9	-	-	1.4
Tetraclinis articulata [205]	440	17	-	4.4	63.5	30.8	18.3	97.7	1.0	53.0	5.0	9.4	30.4	0.9	-	-	1.2
Microbiota decussata [205]	441	19	-	23.0	59.8	14.8	15.9	94.2	3.4	51.9	9.6	13.4	20.9	1.8	-	-	1.1
Diselma archeri [205]	442	21	-	1.8	76.3	21.2	17.8	97.9	1.4	40.4	8.5	12.6	32.5	5.3	-	-	1.5
Cupressus torulosa [205]	443	20	-	0.3	70.4	29.3	18.2	99.6	0.4	29.0	5.2	19.2	43.5	3.1	-	-	1.9
Cupressus dupreziana [205]	444	20	-	1.3	83.9	14.1	17.7	97.4	1.9	29.3	5.2	18.1	44.1	2.6	-	-	1.8
Cupressus sempervirens [208]	445	22	-	1.9	89.5	8.6	17.3	99.2	0.8	29.5	4.3	16.4	48.0	1.8	-	-	1.9
Calocedrus decurrens [205]	446	16	-	tr.	61.6	37.2	18.5	97.7	1.1	39.1	4.5	20.4	30.8	4.0	-	-	1.5
Metasequoia glyptostroboides [205]	447	18	-	3.2	86.3	8.1	17.4	96.6	1.0	24.8	2.9	7.5	59.0	3.4	-	-	2.1
Cryptomeria japonica [205]	448	20	-	0.4	60.2	35.0	18.2	93.0	2.6	30.3	7.5	27.3	27.7	2.8	-	-	1.6
Thuja koraiensis [205]	449	20	-	6.1	74.3	19.1	17.1	95.2	4.3	40.2	11.9	15.9	28.2	3.3	-	-	1.4
Thuja standishii [205]	450	21	-	0.5	80.7	17.8	17.5	90.0	9.0	32.4	11.8	13.1	38.1	3.6	-	-	1.7
Thuja occidentalis [205]	451	18	-	tr.	67.7	30.4	18.3	96.3	1.8	33.8	6.0	19.7	35.2	3.4	-	-	1.7
Thuja plicata [208]	452	22	-	0.9	89.0	10.1	17.7	99.4	0.6	26.0	3.6	16.2	50.7	3.5	-	-	2.0
Cyathea dealbata [212]	453	30	-	0.1	84.2	15.4	17.9	98.9	0.8	30.5	13.4	10.9	35.4	5.5	4.0	-	1.8
Hymenophyllum plicatum [213]	454	16	3.9	1.2	57.4	37.8	17.8	82.0	18.3	16.3	25.2	2.0	49.8	-	-	7.0	2.2
Hymenophyllum caudiculatum [213]	455	15	5.3	1.3	51.4	41.9	17.8	85.6	14.3	13.6	19.4	-	65.5	-	-	1.4	2.2
Ptisana salicina [214]	456	27	-	-	88.1	11.9	17.8	99.6	0.4	25.3	7.3	21.2	38.9	7.1	0.2	-	2.0
Salvinia natans [214]	457	65	-	1.6	66.1	31.1	18.1	92.2	6.6	21.6	27.4	9.1	12.5	14.9	8.4	4.9	2.1
Azolla caroliniana [215]	458	24	-	0.3	86.2	11.1	17.4	96.7	0.9	45.1	13.8	7.7	26.6	4.4	-	-	1.3
Lygodium japonicum [216]	459	22	-	tr.	87.3	13.1	17.9	98.2	2.2	46.8	32.7	15.0	5.9	-	-	-	0.8
Claytosmunda claytoniana [212]	460	32	-	0.1	90.8	9.3	17.5	99.2	1.0	22.5	6.8	6.3	57.6	5.1	1.9	-	2.2
Osmunda regalis [217]	461	19	-	tr.	89.6	10.4	17.6	99.5	0.5	22.3	5.4	5.3	59.0	4.7	3.3	-	2.3
Osmundastrum cinnamomeum [216]	462	23	-	11.7	62.4	25.7	18.8	95.4	4.8	92.98	6.8	0.3	0.3	-	-	-	0.1
Woodsia glabella [218]	463	12	-	-	80.7	16.3	18.1	97.0	-	36.7	14.8	11.0	28.6	5.9	-	-	1.5
Amauropelta noveboracensis [212]	464	28	-	0.1	91.4	8.6	17.7	99.4	0.7	19.1	6.2	7.1	60.4	5.8	1.5	-	2.3
Phegopteris connectilis [212]	465	29	-	-	86.2	13.6	17.6	98.7	1.1	29.9	9.3	7.7	43.3	5.8	3.8	-	2.0
Pyrrosia eleagnifolia [212]	466	29	-	0.6	76.0	23.3	18.1	99.3	0.6	34.2	8.8	18.1	26.8	11.7	0.3	-	1.7
Platycerium bifurcatum [102]	467	14	-	-	89.2	10.8	17.6	99.3	0.7	44.8	15.8	21.8	15.8	1.8	-	-	1.1
Pleopeltis polypodioides [216]	468	21	-	0.1	72.6	26.9	18.9	95.4	4.2	51.8	18.1	28.6	1.1	-	-	-	0.8
Polypodium vulgare [219]	469	16	-	-	74.0	26.0	18.3	100.0	-	27	8	14	36	13	2	-	2.1
Gymnocarpium dryopteris [218]	470	12	-	-	87.9	9.1	17.5	97.0	-	42.8	13.5	7.7	28.4	4.6	-	-	1.3
Cystopteris fragilis [212]	471	28	-	0.1	90.7	9.0	17.6	98.7	1.1	24.3	9.6	22.3	38.8	4.6	0.3	-	1.9
Deparia pycnosora [212]	472	29	-	0.1	88.8	10.8	17.7	98.7	1.0	24.1	7.9	11.8	48.0	5.9	2.0	-	2.1
[]Athyrium sinense [212]	473	26	-	-	88.0	11.7	17.9	99.0	0.7	6.5	3.5	5.7	74.1	6.6	3.3	-	2.8

Athyrium distentifolium [218]	474	12	-	-	86.4	8.9	17.8	95.3	-	29.4	18.4	14.6	29.7	3.2	-	-	1.5
Athyrium spinulosum [212]	475	30	-	0.1	89.4	10.6	17.7	99.4	0.7	20.5	7.6	8.9	55.8	5.1	2.2	-	2.2
Athyrium crenulatoserrulatum [212]	476	29	-	0.1	91.5	8.3	17.6	98.9	1.0	22.8	7.9	11.7	51.0	5.0	1.5	-	2.1
Athyrium yokoscense [212]	477	30	-	0.1	88.4	11.5	17.7	98.8	1.2	22.3	10.5	9.5	50.4	5.5	1.8	-	2.1
Athyrium filix0femina [212]	478	30	-	0.1	90.0	9.8	17.8	99.3	0.6	14.1	10.8	17.9	49.8	4.7	2.6	-	2.3
Pteridium esculentum [212]	479	31	-	0.1	83.3	16.6	17.9	99.6	0.4	27.5	7.9	11.9	43.5	8.2	1.0	-	2.0
Pteridium aquilinum [220]	480	33	-	-	77.7	22.0	18.1	99.4	0.3	34.4	7.7	25.3	19.1	12.2	1.0	-	1.7
Asplenium oblongifolium [212]	481	28	-	0.4	84.2	15.4	17.8	99.6	0.4	25.4	6.1	18.1	41.2	8.9	0.3	-	2.0
Asplenium trichomanes [102]	482	23	-	tr.	95.7	4.3	17.6	99.5	0.5	22.8	30.7	35.9	7.8	2.8	-	-	1.4
Asplenium scolopendrium [41]	483	35	-	tr.	85.2	14.8	17.8	99.3	0.8	29.3	11.2	19.8	29.6	9.5	0.7	-	1.8
Asplenium nidus [102]	484	27	-	0.3	78.6	20.9	18.4	98.3	1.5	54.5	13.1	19.5	10.1	2.5	-	-	0.9
Adiantum pedatum [212]	485	30	-	0.2	89.2	10.5	17.6	99.1	0.8	23.7	9.4	7.4	50.1	6.9	2.3	-	2.1
Polystichum tripteron [212]	486	28	-	-	86.5	13.5	17.5	99.0	1.0	38.4	15.1	14.6	24.7	4.5	2.7	-	1.5
Dryopteris austriaca [221]	487	23	-	1.2	88.6	11.7	17.6	100.6	0.9	26.3	6.4	9.7	50.3	6.7	2.1	-	2.1
Dryopteris expansa [218]	488	12	-	-	85.4	10.0	17.8	95.4	-	34.5	11.9	8.2	37.4	3.4	-	-	1.5
Dryopteris goeringiana [212]	489	31	-	0.1	87.8	12.1	17.7	99.0	1.0	26.3	9.9	9.5	47.3	6.1	0.9	-	2.0
Dryopteris crassirhizoma [212]	490	29	-	0.3	89.7	10.3	17.9	99.5	0.8	11.8	7.1	12.4	60.9	5.9	2.2	-	2.5
Dryopteris filix-mas [221]	491	23	-	0.4	86.2	13.4	17.6	99.8	0.2	26.7	7.0	13.7	43.5	7.2	1.9	-	2.0
Onoclea sensibilis [212]	492	28	-	0.1	92.3	7.6	17.6	99.1	0.9	21.0	6.8	7.4	57.9	5.8	1.1	-	2.2
Matteuccia struthiopteris [222]	493	29	-	-	87.3	12.7	17.7	99.7	0.3	27.3	12.6	15.9	35.3	5.6	3.3	-	1.9
Equisetum fluviatile [217]	494	17	-	tr.	91.8	8.2	17.6	99.5	0.5	19.1	4.1	7.8	65.4	3.6	-	-	2.3
Equisetum variegatum [223]	495	19	-	0.1	98.3	10.7	17.6	108.0	1.1	33.2	7.7	33.1	32.6	2.5	-	-	1.8
Equisetum hyemale [224]	496	10	-	-	94.6	5.2	17.5	99.8	-	24.5	1.1	16.2	56.4	1.6	-	-	2.1
Equisetum scirpoides [223]	497	20	-	0.3	93.9	6.7	17.5	99.9	1.0	31.5	7.8	22.6	36.8	2.2	-	-	1.7
Equisetum arvense [223]	498	19	-	0.2	95.5	4.2	17.3	99.3	0.6	29.7	7.9	8.8	51.2	2.3	-	-	1.9
Psilotum nudum [217]	499	22	-	2.4	89.1	8.5	17.5	99.6	0.4	30.4	14.1	19.6	34.9	0.5	0.5	-	1.6
Riccia fluitans [96]	500	33	-	0.9	64.7	4.2	17.5	67.1	2.0	11.1	13.3	15.2	20.7	8.8	-	-	1.4
Conocephalum conicum [26]	501	37	-	0.7	75.5	23.1	17.8	97.7	1.7	39.1	12.7	8.8	21.6	9.1	8.1	-	1.7
Marchantia polymorpha [26]	502	37	-	0.2	80.2	19.4	17.8	98.5	1.3	28.8	15.1	11.2	29.3	5.0	10.4	-	1.9
Metzgeria pubescens [26]	503	37	-	0.2	84.5	15.4	17.7	98.2	1.9	23.4	5.4	42.8	15.0	13.2	0.3	-	1.9
Ptilidium ciliare [26]	504	37	-	0.2	74.3	25.9	18.3	99.0	1.4	20.0	8.6	31.3	22.7	16.1	1.7	-	2.1
Plagiochila porelloides [26]	505	37	-	0.2	83.0	17.2	17.8	99.2	1.2	22.2	7.7	28.6	26.2	8.9	6.8	-	2.1
Pellia neesiana [96]	506	33	-	1.0	69.2	1.0	17.3	69.4	1.8	18.1	19.5	9.0	19.1	5.5	-	-	1.2
Sphagnum majus [225]	507	10	-	2.6	71.3	14.8	17.9	86.1	-	12.7	10	25.9	22.7	0.2	14.6	-	2.0
Sphagnum nemoreum [226]	508	69	-	2.3	76.9	20.5	18.0	93.6	6.1	23.4	35.7	11.1	17.1	9.1	3.3	-	1.6
Sphagnum squarrosum [226]	509	69	-	2.5	77.1	20.2	17.9	91.9	7.9	25.6	39.5	9.4	15.2	6.9	3.0	-	1.5
Sphagnum magellanicum [225]	510	10	-	3.9	80.0	15.4	17.9	99.2	-	17.4	6.6	22.1	37.7	2.6	12.8	-	2.3
Sphagnum fimbriatum [225]	511	10	-	3.6	81.2	16.6	18.0	101.4	-	18.1	6.4	22.1	38.2	3.8	12.8	-	2.4
Aulacomnium palustre [226]	512	61	-	2.6	79.3	17.4	17.8	91.9	7.4	26.2	34.8	11.5	18.2	8.3	0.3	-	1.5
Tortella tortuosa [227]	513	15	-	-	91.1	8.2	17.6	99.3	-	23.7	16.4	26.4	25.2	5.8	1.8	-	1.8
Philonotis fontana [226]	514	61	-	2.6	81.0	15.2	17.8	92.6	7.2	27.8	31.8	14.4	20.4	5.3	0.1	-	1.4
Entodon schleicheri [26]	515	27	-	-	45.4	53.4	19.0	98.3	0.5	18.7	2.8	7.3	24.4	25.1	20.5	-	2.9
Thuidium assimile [26]	516	37	-	0.2	62.9	36.9	18.6	99.2	0.8	18.9	4.6	17	28.6	24.1	6.8	-	2.6
Calliergon cordifolium [96]	517	33	-	1.4	70.6	0.6	17.3	69.5	3.1	16	14.9	9.6	27.4	4.7	-	-	1.4
Hygrohypnella ochracea [226]	518	67	-	2.6	79.5	17.4	17.9	93.1	6.4	19.6	29.3	10.9	27.9	8.7	3.1	-	1.9
Helodium blandowii [226]	519	66	-	3.5	81.9	14.7	17.8	93.2	6.9	27.7	31.4	15.4	19.3	5.9	0.1	-	1.4
Rhytidium rugosum [26]	520	30	-	-	62.7	36.3	18.6	98.8	0.2	9.7	10.6	22.6	25.5	17.3	13.3	-	2.7
Drepanocladus lycopodioides [96]	521	33	-	1.3	71.2	0.9	17.3	70.7	2.7	20.2	22.6	10.4	16.2	4.0	-	-	1.1
Amblystegium riparium [228]	522	18	-	-	70.8	27.6	18.2	98.4	-	25.5	2.7	25.6	20.9	12.2	11.5	-	2.2
Cratoneuron filicinum [26]	523	30	-	-	61.0	38.3	18.6	99.0	0.3	17.4	8.5	10.5	26.0	16.9	20.0	-	2.8
Pseudanomodon attenuatus [26]	524	29	-	-	57.8	41.6	18.7	98.4	1.0	14.5	8.4	19.4	21.3	28.3	7.5	-	2.6
Neckera pennata [26]	525	30	-	-	67.2	32.0	18.5	98.8	0.4	13.2	10.8	23.4	24.6	19.1	8.1	-	2.5
Ctenidium molluscum [227]	526	15	-	-	58.3	39.6	18.3	97.9	-	20.3	7.3	18.3	23.8	22.9	5.3	-	2.3
Climacium dendroides [226]	527	60	-	3.6	80.9	15.2	17.8	90.5	7.2	28.8	31.3	14.3	20.1	5.1	0.1	-	1.4
Fontinalis antipyretica [229]	528	26	-	-	71.9	15.5	18.3	87.4	-	-	3.7	12.5	48.6	18.8	3.6	0.2	2.7
Hypnum andoi [230]	529	6	-	-	71.6	28.4	17.9	100.0	-	87.6	12.4	-	-	-	-	-	0.1
Hypnum jutlandicum [231]	530	14	-	-	69.9	30.1	18.9	98.9	1.1	51.3	33.9	6.5	5.8	2.5	-	-	0.8
Hypnum cupressiforme [232]	531	24	2.1	13.3	76.1	8.5	16.14	90.7	9.3	43.6	23.9	14.9	14.3	3.3	-	-	1.1
Rhytidiadelphus squarrosus [233]	532	10	-	-	47.8	43.3	18.5	91.1	-	19.3	3.9	5.9	23.6	32.7	5.7	-	2.5
Kindbergia praelonga [234]	533	2	-	-	100.0	-	16.2	100.0	-	100.0	-	-	-	-	-	-	-
Eurhynchium praelongum [228]	534	17	-	-	65.8	31.7	18.14	97.5	-	32.8	2.1	12.4	31.5	7.1	11.6	-	2.1
Eurhynchium striatum [233]	535	10	-	-	60.7	40.5	18.4	101.2	-	20.9	5.8	18.3	19.2	34.1	2.9	-	2.5
Rhynchostegium murale [228]	536	19	-	-	61.9	35.9	18.6	97.8	-	25.6	13.6	17.4	21.3	13.5	6.4	-	2.0
Brachythecium erythrorrhizon [228]	537	17	-	-	67.7	31.6	18.4	99.3	-	26.5	2.7	20.7	22.7	13.9	12.8	-	2.3
Brachythecium rivulare [26]	538	31	-	-	49.2	44.8	18.7	93.3	0.7	18.5	6.1	10.0	18.2	19.3	21.9	-	2.7
Brachythecium rutabulum [231]	539	13	-	-	81.2	15.5	17.4	94.9	1.8	42.7	22.6	7.5	10.6	7.9	5.4	-	1.3
Calliergonella cuspidata [235]	540	6	-	-	86.7	3.3	16.6	90.0	-	71.8	8.2	6.1	3.9	-	-	-	0.3
Bryum sp . [226]	541	66	-	2.7	79.3	18.0	17.9	93.5	6.5	19.9	29.7	10.5	28.2	8.6	3.1	-	1.9

Ptychostomum moravicum [231]	542	12	-	-	74.2	25.8	18.2	100.0	-	37.9	15.8	10.3	19.6	12.2	4.2	-	1.7
Ptychostomum pseudotriquetrum [226]	543	65	-	2.0	76.4	21.3	18.2	93.7	5.7	18.2	26.5	13.3	27.5	11.8	2.1	-	1.9
Rhodobryum ontariense [236]	544	8	-	-	100.0	-	17.7	100.0	-	15.5	2.4	5.3	34.6	42.2	-	-	2.9
Pseudobryum cinclidioides [226]	545	61	-	3.9	80.4	16.7	17.7	93.3	6.7	21.5	37.1	13.6	20.3	7.3	0.2	-	1.6
Plagiomnium confertidens [26]	546	37	-	0.2	55.3	41.8	18.7	96.5	0.8	27.8	7.0	7.6	20.9	26.5	7.5	-	2.3
Plagiomnium medium [226]	547	66	-	2.7	79.3	17.9	17.9	92.1	7.8	19.8	29.6	10.5	28.4	8.5	3.1	-	1.9
Plagiomnium cuspidatum [237]	548	8	-	-	60.2	39.8	18.3	100.0	-	25.7	9.0	8.3	17.2	33.8	6.0	-	2.4
Mnium hornum [238]	549	7	-	-	64.5	32.5	18.2	97.0	-	27.9	5.1	11.8	19.7	26.1	6.4	-	2.2
Ceratodon purpureus [239]	550	15	-	-	83.2	13.1	17.7	96.3	-	17.8	5.9	14.9	44.2	11.7	1.8	-	2.2
Dichodontium pellucidum [227]	551	15	-	-	88.2	9.5	17.8	97.7	-	10.0	8.6	37.0	37.5	3.5	1.1	-	2.1
Funaria hygrometrica [240]	552	8	-	-	82.7	-	16.9	82.7	-	32.4	25.3	20.6	4.4	-	-	-	0.8
Physcomitrium patens [24]	553	13	-	-	70.6	29.4	18.0	100.0	-	36.5	0.3	23.4	19.6	18.7	1.5	-	1.9
Pogonatum urnigerum [227]	554	15	-	-	17.7	81.6	19.5	99.3	-	4.2	1.3	8.1	81.8	3.5	0.4	-	2.8
Atrichum undulatum [230]	555	8	-	-	91.3	8.7	17.8	100.0	-	26.5	18.5	26.8	20.5	6.2	1.5	-	1.7
Polytrichum juniperinum [226]	556	64	-	2.3	75.9	21.1	18.0	92.2	7.1	23.5	33.8	12.1	17.3	9.2	3.4	-	1.6
Polytrichum commune [26]	557	29	-	-	73.4	25.8	18.2	98.8	0.4	21.3	5.2	14.7	40.6	14.5	2.9	-	2.3

* – the first publisher data
