# Supplementary figures and images for: Phylogenetic Aspects of Higher Plant Lipid Fatty Acid Profile
Source: Int J Mol Sci. 2025 Sep 26;26(19):9424. doi: 10.3390/ijms26199424 (PMC12524838; doi:10.3390/ijms26199424)

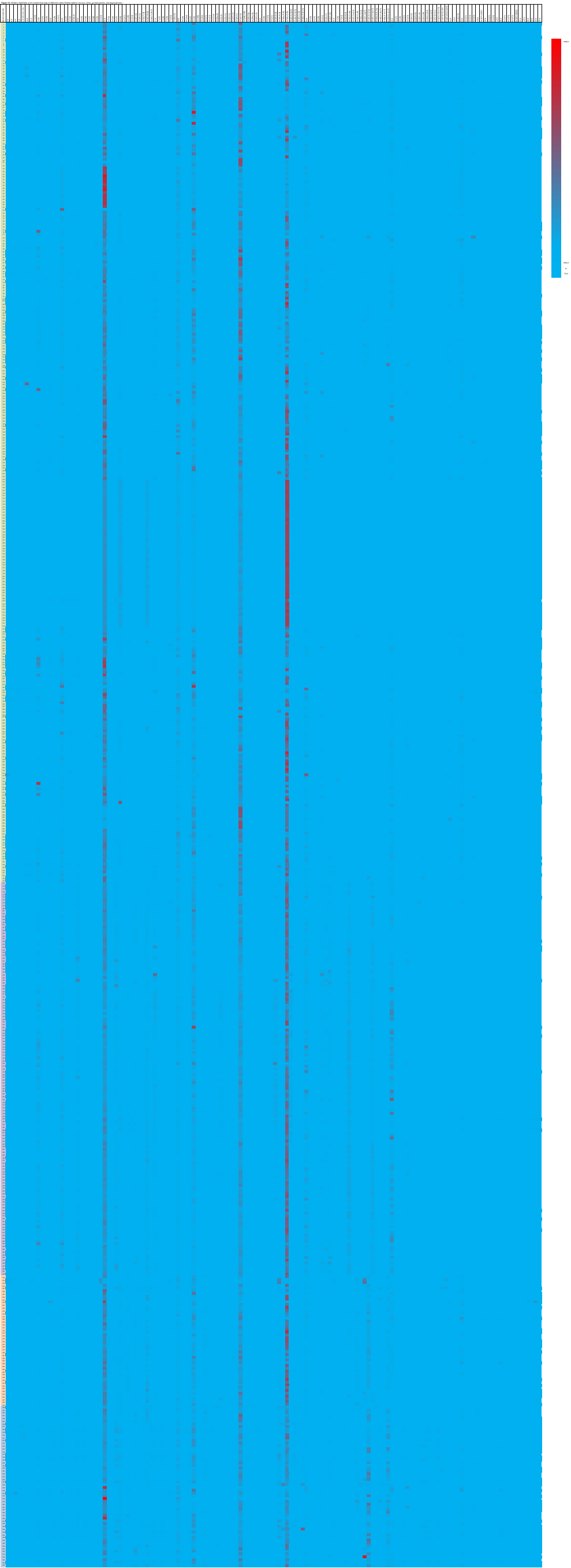

Supplement: Supplementary file 1 [file ijms-26-09424-s001.zip › Figure_S1.pdf]
